# MEF-AlloSite: an accurate and robust Multimodel Ensemble Feature selection for the Allosteric Site identification model

**DOI:** 10.1186/s13321-024-00882-5

**Published:** 2024-10-23

**Authors:** Sadettin Y. Ugurlu, David McDonald, Shan He

**Affiliations:** 1 School of Computer Science, University of Birmingham, Edgbaston, Birmingham, B15 2TT UK; 2 AIA Insights Ltd, Birmingham, UK

**Keywords:** Allosteric binding site, Allostery, Binding site, Multimodel Ensemble Feature selection

## Abstract

A crucial mechanism for controlling the actions of proteins is allostery. Allosteric modulators have the potential to provide many benefits compared to orthosteric ligands, such as increased selectivity and saturability of their effect. The identification of new allosteric sites presents prospects for the creation of innovative medications and enhances our comprehension of fundamental biological mechanisms. Allosteric sites are increasingly found in different protein families through various techniques, such as machine learning applications, which opens up possibilities for creating completely novel medications with a diverse variety of chemical structures. Machine learning methods, such as PASSer, exhibit limited efficacy in accurately finding allosteric binding sites when relying solely on 3D structural information.

**Scientific Contribution**

Prior to conducting feature selection for allosteric binding site identification, integration of supporting amino-acid–based information to 3D structural knowledge is advantageous. This approach can enhance performance by ensuring accuracy and robustness. Therefore, we have developed an accurate and robust model called Multimodel Ensemble Feature Selection for Allosteric Site Identification (MEF-AlloSite) after collecting 9460 relevant and diverse features from the literature to characterise pockets. The model employs an accurate and robust multimodal feature selection technique for the small training set size of only 90 proteins to improve predictive performance. This state-of-the-art technique increased the performance in allosteric binding site identification by selecting promising features from 9460 features. Also, the relationship between selected features and allosteric binding sites enlightened the understanding of complex allostery for proteins by analysing selected features. MEF-AlloSite and state-of-the-art allosteric site identification methods such as PASSer2.0 and PASSerRank have been tested on three test cases 51 times with a different split of the training set. The Student’s t test and Cohen’s D value have been used to evaluate the average precision and ROC AUC score distribution. On three test cases, most of the p-values ($< 0.05$) and the majority of Cohen’s D values ($> 0.5$) showed that MEF-AlloSite’s 1–6% higher mean of average precision and ROC AUC than state-of-the-art allosteric site identification methods are statistically significant.

**Supplementary Information:**

The online version contains supplementary material available at 10.1186/s13321-024-00882-5.

## Introduction

The linkage of conformational changes between two physically distant locations is known as allostery. It has been referred to as “the second secret of life” and is one of the most popular and effective ways to control protein activity [1]. An allosteric site is topographically distinct from an orthosteric site. In contrast to orthosteric active site inhibitors, allosteric binding sites show more sequence variability across protein subtypes, enabling the development of more selective ligands, which results in higher allosteric site structural diversity [2]. For drug design, there are several benefits to the higher allosteric site structural diversity, such as enhanced subtype selectivity, decreased drug resistance, low toxicity, and the capacity to specifically tune (activate or inhibit) the response of the target protein [3–5]. As a result of these benefits, the variety of methods for identifying allosteric sites has steadily increased in recent years, such as experimental approaches and in silico methods [6, 7].

Experimental approaches, including high-throughput screening [8], fragment-based screening [9], and disulfide trapping [10], encounter difficulties due to the rapid increase in the number of allosteric drug targets, as well as the limited ability of biassed chemical libraries to identify possible allosteric sites. Alternatively, in silico methods that offer fast platforms for discovering allosteric regions in proteins have been acknowledged as valuable tools [6]. Several in silico methods fall under five main categories: (i) molecular dynamics (MD)-based prediction, (ii) normal-mode-analysis (NMA)-based prediction, (iii) combination of dynamics- and NMA-based prediction, (iv) sequence-based prediction, and (iv) structure-based prediction, have been created to forecast allosteric sites [11–20].

Molecular dynamics (MD) simulations utilise a comprehensive model of interatomic interactions to forecast the movement of each atom inside a protein or other molecular system over time [21]. For example, two-state G models [17] and Markov state models [16] have been used to identify allosteric binding sites. A coarse-grained two-state Gō model is formed by combining two individual single-state Gō potentials [22], such as the T (tense) and R (relaxed) states in allostery. The T and R states in allostery are two distinct conformations of an allosteric protein. The T state is generally characterised by reduced activity or inactivity and exhibits a lower affinity for the ligand or substrate. In contrast, the R state is more active and demonstrates a higher affinity for the ligand or substrate. The transition between these states is crucial for the regulation of the protein’s function, enabling it to react to various signals or alterations in the cellular environment [3, 23, 24]. Also, Markov state modelling tools for proteins are computational methods that analyse and explain protein dynamics by dividing the conformational space into distinct states and calculating the odds of transitioning between these states over time. These models offer a conceptual structure for comprehending the extended temporal patterns of proteins, enabling researchers to anticipate their kinetic and thermodynamic characteristics [15]. The anticipation can indicate an allosteric site.

Normal Mode Analysis (NMA) is a straightforward computational method for estimating the flexibility of protein structures. The change in flexibility resulting from the binding of a ligand to a specific position in the protein structure has been employed to identify allosteric binding sites [15]. For instance, the Protein Allosteric and Regulatory Sites (PARS) web server [14], developed by Panjkovich and Daura, utilise NMA to predict the precise locations of allosteric sites in proteins by examining the changes in protein flexibility caused by ligand binding [14, 15].

The integration of dynamics- and NMA-based approaches have been employed to enhance the resilience and efficiency of identifying allosteric binding sites. One of the most common examples of the method is SPACER [13], which performed Monte Carlo simulations to explore the protein’s surfaces. During the simulation, the strain on ligand–protein interactions at each potential location is assessed using low-frequency normal modes. Once the ligand interacts with residues that move in opposite directions, significant strain is caused, and this location exhibits a high level of binding leverage. Population shift can lead to significant alterations in protein structure when ligands bind to an allosteric site. As a result, SPACER can identify the allosteric binding site [13].

Several sequence-based in silico methods are available to identify the allosteric binding site, such as Mutual Information (MI) analysis [25], Statistical Coupling Analysis (SCA) [26], Direct Coupling Analysis (DCA) [25, 26], and Multiple Sequence Alignments (MSAs) [26]. MI analysis is a method used to detect allosteric binding sites in proteins. It does this by quantifying the statistical relationship between different places in the protein sequence. This allows for the identification of co-evolving residues that are potentially involved in allosteric control [27]. The SCA method identifies allosteric binding sites by measuring the evolutionary restrictions on certain amino acid locations. This allows for the identification of networks of residues that co-evolve and may impact allosteric communication [26, 28–31]. DCA detects allosteric sites by directly deducing the pairwise connections between residues from a multiple sequence alignment, emphasising the contacts that play a role in the allosteric control [32]. MSAs facilitate the identification of allosteric binding sites by matching sequences from homologous proteins to identify conserved and variable areas. Thus, the residues that play a significant role in allosteric activity are pinpointed [33].

As for the structure-based allosteric site identification, Huang et al. [12] identified 90 distinct allosteric sites from Allosteric Database v2.0 (ASD) [34]. They used these sites to create a server-based model called AlloSite that accurately predicts allosteric sites. AlloSite utilises Fpocket to detect pockets and generate 19 features, which are then employed to train the support vector machine (SVM) classifier. More recent studies used the similar approaches are PASSer [4, 5], PASSer2.0 [35], PASSerRank [36], and P2Rank [37, 38]. The structure-based approach requires less computational power and time compared to MD-based, NMA-based, and a combination of the two. Additionally, it can offer superior performance compared to sequence-based methods, although structure-based methods have inherent limitations in terms of their performance.

**Fig. 1:**
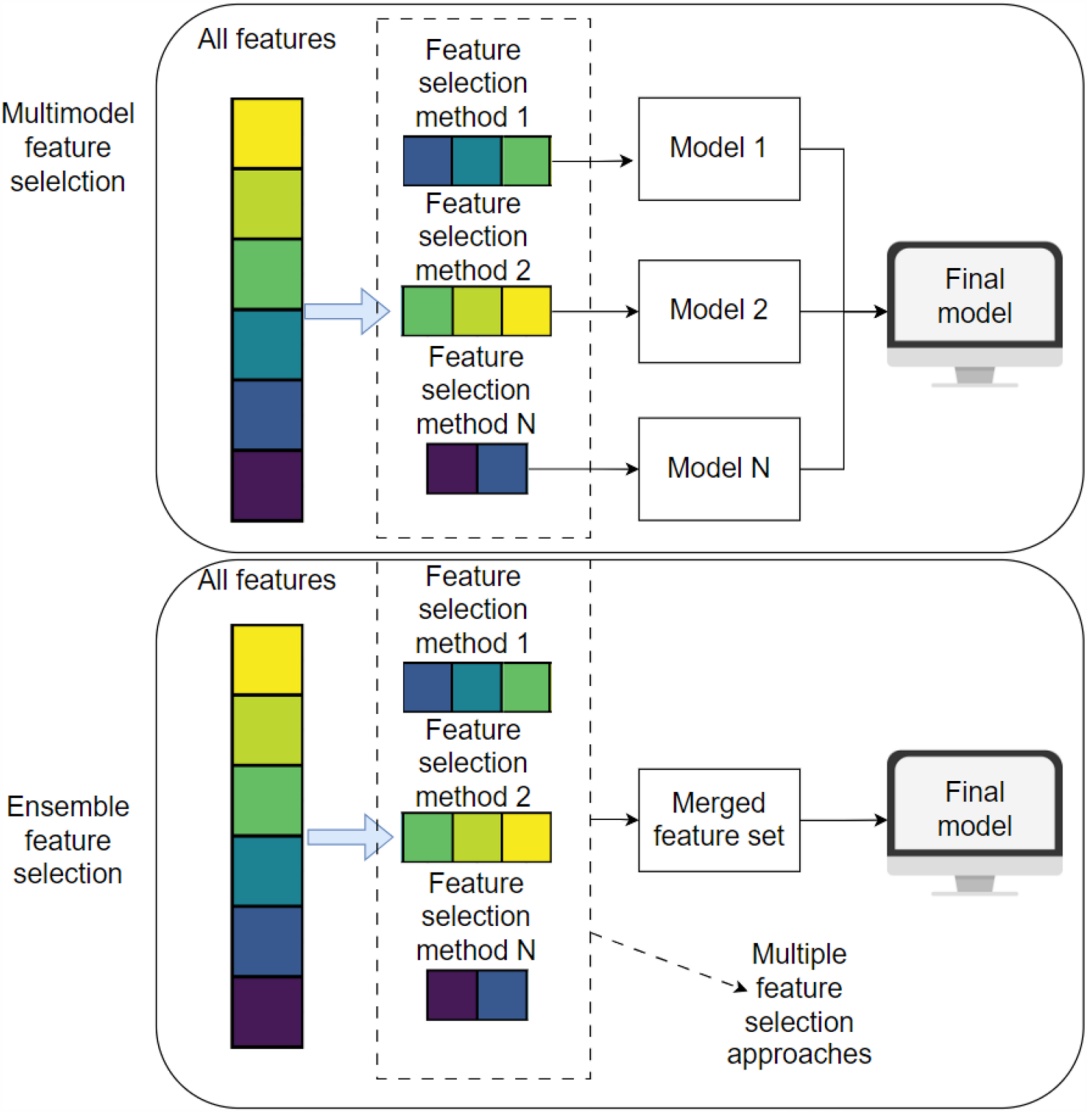
Comparative Illustration of Multimodel and Ensemble Feature Selection Approaches. The upper section illustrates the multimodel feature selection process, where diverse feature selection methods generate different subsets of features. These subsets are then used to train multiple models, and the outputs of these base models are linearly weighted to produce the final prediction. The lower section demonstrates the ensemble feature selection approach, which also employs various feature selection methods. However, instead of training separate models on different subsets, it merges the outputs into a single, unified feature set. This consolidated feature set is subsequently used to train the final model, streamlining the feature selection process and potentially enhancing model performance

**Fig. 2:**
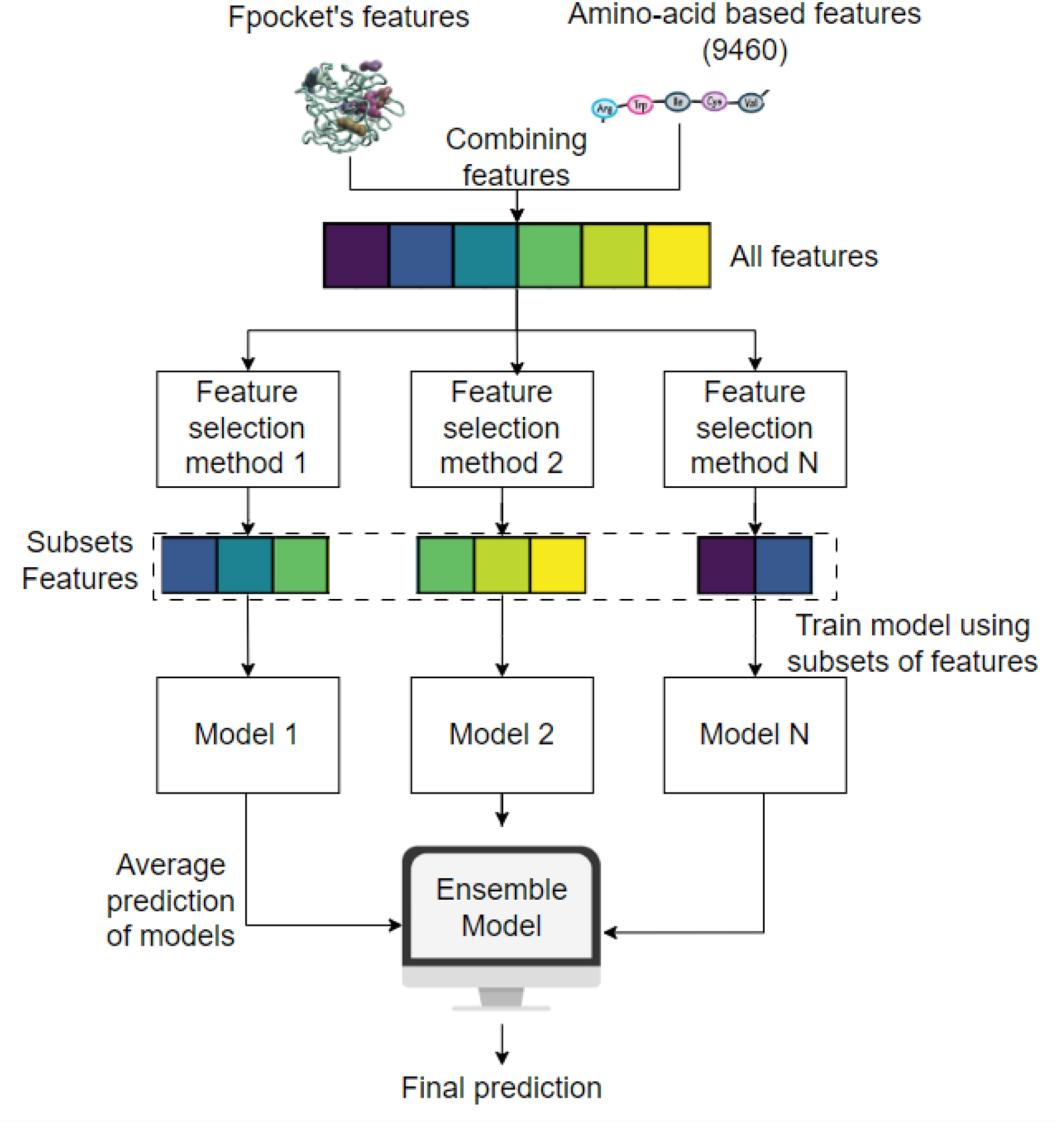
The graphic presents a visual representation of the architectural improvements of the MEF-AlloSite concept. MEF-AlloSite utilises 3D structural information and amino acid–based characteristics to incorporate 9460 features at the start of the process. Then, the N feature selection approach can detect distinct patterns within the dataset. The N feature selection approach has been employed to identify N sets of features. The N feature set has been employed to train N models using AutoGluon, which serves as the base model. The process of aggregating model predictions involves the linear weighting of N models. Each model’s prediction probability has been utilised once to get the average forecasts. Hence, the application of linear weighting to the base model holds the potential to yield improved performance compared to the individual performance of each model

Many existing structural-based tools have been trained with a limited number of features, often derived from cavity detection tool Fpocket [4, 5, 12, 38–41]. However, despite some success, the 19 Fpocket-derived features are insufficient to capture allostery and understand the mechanism of protein allostery without utilizing additional amino acid–based features. Amino acid–based features possess the capacity to provide valuable insights into the organisation and composition of components within a certain cavity structure. The information contained in the constitution has practical use in predicting the behaviour or properties of the cavity [42, 43]. Moreover, the utilisation of amino acid–based characteristics has the potential to be employed in the grouping of cavities [44, 45]. Additionally, it is crucial to consider both structural and amino acid–based data to elucidate the fundamental functions of proteins. These two types of information offer complimentary perspectives that have yet to be thoroughly explored [46]. By incorporating amino acid–based features with Fpocket-derived features, the overall diversity of features is increased. The high diversity of beneficial features plays a crucial role in developing an accurate and robust machine-learning model for identifying allosteric binding sites. However, the inclusion of both structural and amino acid–based data may provide a curse of dimensionality due to small numbers of known allosteric binding pairs, resulting in limited training sample numbers. Hence, there is a possibility for further inquiry into examining a wider range of features and developing an efficient algorithm to identify allosteric sites. An efficient algorithm can employ a multimodal feature selection technique to provide increased resilience, higher accuracy, and the capability to capture intricate connections by overcoming the curse of dimensionality (Figs. 1, and 2).

To develop an accurate and robust model for identifying allosteric binding sites, a total of 9460 pertinent features were gathered from a range of sources within the scientific literature. However, naively using all the features from all of these sources has the drawback that many features may be redundant or extraneous—for example, features that pertain to the pocket rather than the allosteric binding site. Using multimodel feature selection techniques exhibits considerable potential in augmenting the efficacy of machine learning models by eliminating extraneous features [7, 47–50]. Therefore, multimodel feature selection has been used to increase the performance of allosteric binding site identification by overcoming the curse of dimensionality challenge [2, 12, 51]. The recently announced Multimodel Ensemble Feature selection strategy provided to overcome the curse of dimensionality for the small size of the training sets. It also provides notable benefits in terms of improved and consistent performance by leveraging multiple feature selection strategies simultaneously [47]. The Multimodel Ensemble Feature selection technique selects multiple feature subsets to train base models. The base model outputs have been averaged to find the output of the ensemble model [47]. Identifying allosteric binding sites is significantly facilitated by utilising diverse sources and incorporating a novel feature selection methodology. Consequently, the proposed model is called Multimodel Ensemble Feature selection for the Allosteric Site identification (MEF-AlloSite) to achieve an accurate and robust performance. The MEF-AlloSite pipeline is freely and publicly available for academic use: https://github.com/yauz3/MEF-AlloSite.

## Materials and methods

MEF-AlloSite combines both 3D structural and amino acid–based pocket features in order to improve the performance of allosteric binding site identification compared to purely structural-based approaches, such as PASSer (PASSer website) [5]. However, leveraging both types of features drastically increases the total feature number, which could cause models to be negatively affected by the curse of dimensionality, especially when so few allosteric sites can be used for model training. As a result, a well-designed and robust feature selection approach for a small training set is necessary to improve the performance in allosteric binding site identification significantly. Therefore, To obtain robust and high-ranking performance, various feature set selection methods have been used to select subset features for training models and then linearly weighted models to construct an ensemble model (Fig. 2).

The construction and evaluation of MEF-AlloSite consist of seven main steps: (i) Pocket Identification, (ii) Integrating 3D Structural Data with Amino Acid Features, (iii) Multimodel Ensemble Feature selection, (iv) Model Construction using AutoGluon, (v) Preparing test sets, (vi) Comparison with State-of-art Methods and (vii) Performance Evaluation and Statistical Tests. Each step will be introduced in detail below.

### Pocket identification

Multiple pocket identification programs are documented in the literature (Table 17). Fpocket is the most used pocket identification tool due to its numerous benefits despite its decade of existence. Fpocket offers distinctive functionalities to enhance performance, like charge and volume scores. It is also a simple, quick, and precise standalone tool that is highly suited for an automated workflow. Following PASSer2.0 and AlloPred, Fpocket was employed to detect and characterise possible binding sites within protein structures. Subsequently, the model reranks the pockets found by Fpocket based on their suitability for allosteric binding.

Prior to detecting cavities on proteins using Fpocket, extraneous components present in PDB files, including water molecules, free ions, free atoms, and bound ligands, were eliminated. Then, the proteins lacking an allosteric binding site were removed according to the protocol of PASSer2.0.

The cavities identified by Fpocket may include incomplete residues. However, using residue completion to correct cavities may lead to a more accurate depiction of those cavities by using a complete amino acid sequence. Therefore, residues on found cavities and complete residue atoms have been determined. Consequently, incompletely resolved residues have been included in the protein’s cavity PDB file. The completion process only impacts the generation of amino acid–based features since the Fpocket features are preserved before completion.

As a summary, Fpocket has been used to determine the binding site, following the approaches, including PASSer [4, 5] PASSer2.0 [35], PASSerRank [36], and AlloPred [39]. The Fpocket parameters used for pocket identification were identical to those employed by PASSer2.0 [35] and PASSerRank [36]. Therefore, performance improvement has to originate from feature selection and combining 3D structural and amino-acid–based knowledge.

### Integrating 3D structural data with amino acid–based features

Fpocket is a tool used to detect and analyse pockets in protein structures, providing detailed information using a three-dimensional structural perspective. In order to incorporate this analysis of the structure with amino acid-related knowledge, the review of the current literature has been investigated to discover appropriate approaches. As a result, it was decided to examine the techniques specified in both Table 1 and Table 16 (Supplementary Information). The criteria used had two primary aspects: firstly, the approach needed to be suitable for short sequences, and secondly, its results should not be influenced by the order of amino acid sequences to have high reproducibility. Specifically, certain techniques, such as the one demonstrated in Table 16, necessitate lengthier sequences to be fully executed, making them inappropriate for our objectives. Otherwise, insufficiently lengthy amino acid inputs resulted in the absence of any feature for pockets. Also, the alteration of residue order by Fpocket may potentially impair the functionality of the feature tool that relies on the specific amino acid order, possibly leading to inaccuracies or errors. To ensure reproducibility and suitability of usage for short sequences, the tools shown in Table 16 have not been involved in the pipeline of MEF-AlloSite. As a result, Table 1 shows the 9460 amino acid based-features under consideration in addition to Fpocket features.

**Table 1:** A review of techniques and sub-techniques for the analysis of binding pockets

Method	Submethod	Source
Fpocket	Fpocket	Fpocket
CTD	Composition	PyBioMed
	Composition charge	PyBioMed
	Composition hydrophobicity	PyBioMed
	Composition normalized VDWV	PyBioMed
	Composition polarity	PyBioMed
	Composition polarizability	PyBioMed
	Composition secondary Str	PyBioMed
	Composition solvent accessibility	PyBioMed
	Distribution	PyBioMed
	Distribution charge	PyBioMed
	Distribution hydrophobicity	PyBioMed
	Distribution normalized VDWV	PyBioMed
	Distribution polarity	PyBioMed
	Distribution polarizability	PyBioMed
	Distribution secondary Str	PyBioMed
	Distribution solvent accessibility	PyBioMed
	Transition	PyBioMed
	Transition charge	PyBioMed
	Transition hydrophobicity	PyBioMed
	Transition normalized VDWV	PyBioMed
	Transition polarity	PyBioMed
	Transition polarizability	PyBioMed
	Transition secondary Str	PyBioMed
	Transition solvent accessibility	PyBioMed
Biopython	General features (e.g, MW	Biopython
QuasiSequenceOrder module	Quasi Sequence Order	PyBioMed
	Quasi Sequence Order1	PyBioMed
	Quasi Sequence Order1 Grant	PyBioMed
	Quasi Sequence Order 1SW	PyBioMed
	Quasi Sequence Order2 Grant	PyBioMed
	Quasi Sequence Order2 SW	PyBioMed
	Quasi Sequence Orderp	PyBioMed
	Sequence order coupling number	PyBioMed
	Sequence order coupling number grant	PyBioMed
	Sequence order coupling number SW	PyBioMed
	Sequence order coupling number total	PyBioMed
	Sequence order coupling numberp	PyBioMed
K-Gap	K-Gap	MathFeature

This table displays a range of techniques and sub-techniques used to analyse and describe binding pockets in molecular structures. The methods encompass Fpocket, CTD, Distribution, Transition, Biopython, QuasiSequenceOrder module, and K-Gap. Each method provides distinct methodologies to examine the composition, distribution, transition, general characteristics, sequence order, and other attributes of binding pockets

Table 1 presents a comprehensive overview of the elements designed to integrate 3D structural knowledge with amino acid–based information. For example, Fpocket offers fundamental 3D structural knowledge by detecting probable binding sites through analysis of cavity shapes and sizes. On the other hand, the CTD (Composition, Transition, and Distribution) descriptors provided by PyBioMed provide a more comprehensive analysis of the chemical characteristics of these pockets (Table 1). As an illustration, the Composition Hydrophobicity metric specifically identifies the existence of hydrophobic amino acid residues, which are essential for comprehending the interactions with non-polar ligands. Transition measures, such as Transition Charge, provide insight into the alteration in charge distribution within the pocket, which is crucial for determining binding affinity. Moreover, the QuasiSequenceOrder module effectively captures the sequential organisation of residues, providing valuable insights into the structural context that may be overlooked by mere compositional data (Table 1). Also, QuasiSequenceOrder1 examines the impact of adjacent residues, which can have a substantial effect on the dynamics of the binding site (Table 1). The K-Gap approach from MathFeature analyses the distance between particular residues, offering a distinct viewpoint on the geometric limitations of the binding site (Table 1).

In summary, after excluding the feature sets listed in Table 16, a grand total of 9460 features were selected to reflect the amino acid characteristics of pockets. The 9460 features obtained using the methods described in Table 1 are significantly large considering the training set size of 90 proteins for AlloSite [12]. Due to the presence of more than 9000 characteristics and a limited training set of just 90 known high-quality allosteric binding pairs, feature selection is employed to overcome the curse of dimensionality.

### Multimodel Ensemble Feature selection

Feature selection is an essential preprocessing step in machine learning and data analysis. Its primary objective is identifying and extracting the most relevant and informative features from a given dataset. The dimensionality of contemporary datasets is progressively increasing, necessitating the selection of an optimal subset of features to enhance model performance, mitigate overfitting, and better comprehend the underlying data patterns. Hence, MEF-AlloSite utilised a state-of-the-art method called Multimodel Ensemble Feature selection to improve performance in allosteric binding site identification. Subsequently, selected features using Multimodel Ensemble Feature selection have been examined in order to gain a comprehensive understanding of the correlation between these features and protein allostery.

#### Multimodel Ensemble Feature selection

**Fig. 3:**
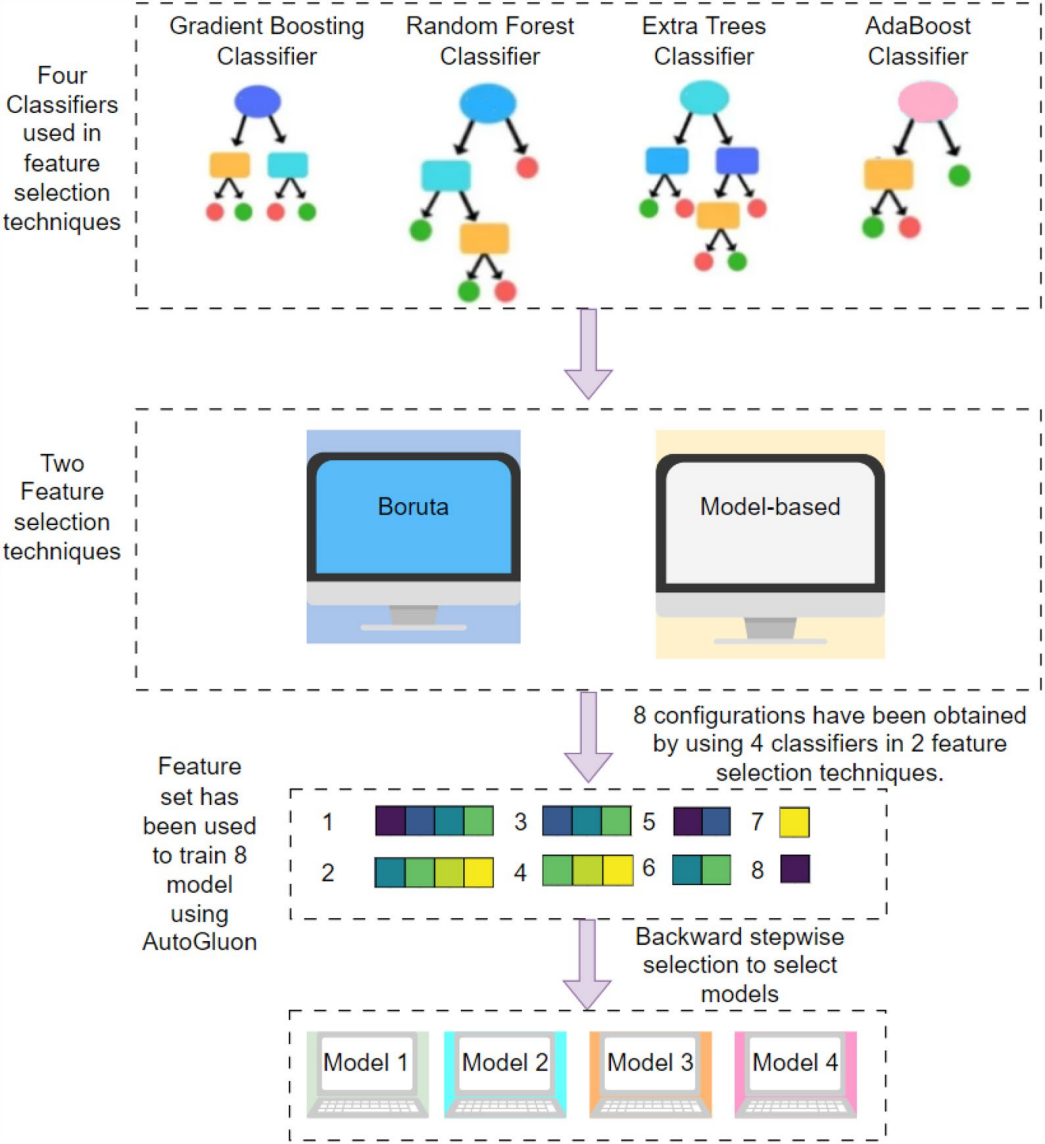
The schematic representation of Multimodel eensemble Feature selection. The utilisation of tree-based classifiers, including Gradient Boosting, Random Forest, Extra Trees, and AdaBoost Classifier, has been implemented in two concurrent feature selection techniques, namely (i) Boruta and (ii) Model-based feature selection. The Boruta algorithm employs a classifier as an estimator and subsequently optimises the number of features. The default classifier was utilised to generate four feature sets through the Boruta feature selection methods. Additionally, each model has been employed to rank features according to their importance, followed by applying forward stepwise selection to determine the number of features. Subsequently, a total of eight feature sets (obtained using two model-based feature selection techniques, each based on four classifiers) have been generated by combining two parallel pipelines, thereby facilitating the accomplishment of Multimodel Ensemble Feature selection. In the final stage, the technique of backward step-wise selection was employed to optimise the number of features in the set. Finally, four models were chosen for MEF-AlloSite. The outputs of the four models have been combined using linear weights to get a final prediction

The concept behind ensemble feature selection methods is to utilise many feature selection models and combine their outputs to enhance the performance of a model (Fig. 1). The difference between multimodel and ensemble feature selection is the method of aggregating features from different feature selection methods. Ensemble feature selection utilises numerous feature selection techniques and merges them in the early stage to create a single consensus-based subset upon which the final model is trained (Fig. 1). On the other hand, Multimodel Ensemble Feature selection involves training individual models for each feature set, which are then combined into the final ensemble model as a secondary layer of model architecture (Fig. 1). The construction of ensemble and Multimodel Ensemble Feature selection has included using two feature selection techniques: Boruta and model-based feature selection (Fig. 3).

A multimodel feature selection approach was employed using eight distinct methods, combining four tree-based classifiers with two feature selection techniques: Boruta and model-based feature selection (Fig. 3). The rationale for this strategy is rooted in the complementary strengths of these methods. Tree-based classifiers, known for their robustness and ability to handle complex, non-linear relationships, provide diverse perspectives on feature importance. Therefore, tree-based classifiers have been used in Boruta and Model-based feature selection techniques. Boruta, an all-relevant feature selection method, ensures that no potentially important feature is overlooked by comparing random and actual feature relevance. Meanwhile, model-based feature selection emphasizes the features that directly contribute to the model’s predictive performance. By integrating these approaches, the goal is to capture a comprehensive set of relevant features, enhancing the robustness and generalizability of the model. Such a multimodel feature methodology leverages the strengths of each technique, thereby improving the likelihood of identifying the most informative features and increasing the predictive accuracy of the final model (Fig. 3).

Eight subsets of 9460 features were selected using two feature selection techniques (Fig. 3), including Boruta and Model-Based Feature selection techniques. Four distinct classifiers have been employed to implement the Multimodel Ensemble Feature selection technique in these two techniques. After using four classifiers and two feature selection approaches, eight feature sets have been obtained (Fig. 3). AutoGluon was utilised to train eight distinct models employing a total of eight feature sets. The backwards stepwise selection starting from 8 feature sets has been used to optimise feature set numbers (Fig. 3). The backward section was employed to optimise the numerical feature set. Initially, all the model coefficients have been assigned a value of 1. Then, each model is discarded to construct an ensemble model, tested on 51 different validations (20% of the training set) and then averaged 51 performance metrics. The largest improvement from discarding a component has been kept for the next iteration. Consequently, the process was stopped when the ensemble model had the highest performance. Ultimately, four models demonstrated the highest level of performance when used in the ensemble model structure. Consequently, the feature sets Feature 1, 2, 3, and 4 were selected to train the models Model 1, 2, 3, and 4, respectively by using AutoGluon. These models were then employed to implement multimodel feature selection and ensemble feature selection procedures. The four selected feature sets and feature sets have been analysed to understand the mechanism of protein allostery (Fig. 3).

To summarize, the study utilized eight different feature selection approaches, integrating four tree-based classifiers with two feature selection techniques, Boruta and model-based feature selection strategies. A reverse search method was employed to identify four optimal subsets from the original eight. The four chosen subsets were used to train a base model for MEF-AlloSite by using AutoGluon. Then, the outputs of the base model have been linearly weighted to obtain the final output for MEF-AlloSite. As for the construction of the ensemble feature selection method (Fig. 1), the four feature subsets were merged to train the Ensemble Features Model (Fig. 1). Consequently, the performance of these two advanced feature selection methods in identifying allosteric binding sites has been examined.

#### Analysis of features

Understanding the complex connection between characteristics and allosteric processes is crucial in identifying allosteric binding sites in proteins. Examining characteristics is also crucial for understanding the intricate interaction between structural and functional components that control allosteric regulation. Therefore, selected features in four feature sets have been examined to comprehend the intricate phenomenon of protein allostery.

Feature analysis is a thorough evaluation of the importance and impact of features on the overall predictive capability of a model. An integral element of this research is determining the frequency of feature selection, which evaluates how frequently a certain feature is picked by the chosen four feature selection algorithms. The four chosen feature sets, namely Feature 1, 2, 3, and 4, have been combined to conduct a more in-depth examination of these features. Furthermore, methods such as ANOVA F importance and correlation matrix analysis are essential for assessing the significance and connections among merged features. The ANOVA F importance evaluates the significance of each characteristic by examining the variation among different groups or classes in the sample. Correlation matrix analysis helps to understand the relationships between different features, revealing possible concerns of multicollinearity and providing guidance for selecting the most appropriate features. Such a feature analysis not only improves the comprehensibility of models by providing insight into their functioning but also adds an additional understanding of protein allostery in the specific field of study.

### Model construction using AutoGluon

MEF-AlloSite’s model construction procedure consists of two essential steps: (i) Preparation of the training set and (ii) Training the model. Initially, the data is carefully selected and prepared to guarantee that the model receives input of excellent quality. In the second step, advanced approaches are utilised to train the model and improve its performance for accurate predictions.

#### Training set preparation

The Allosteric Database (ASD) [34] was utilised in this study to both train and evaluate the predictive power of all machine learning models. Its most recent edition of ASD contains 1949 target entries, each with a unique protein and modulator.

ASD has 1949 protein data, yet its inconsistent data resolution is rather troublesome, particularly for theorists, since the inconsistent data directly impacts a model performance [52], which can reduce the generalisation performance of the model. To guarantee data consistency, however, data from ASD must be filtered according to certain criteria Zha et al. [52]. Therefore, Huang et al. [35] selected 90 proteins to ensure protein quality and variety by following the guidelines. According to the guidelines, there are two main filters: (i) protein structures that either lacked allosteric site residues or were captured at a higher resolution than 3 Å should be removed, and (ii) the remaining data should be filtered to remove redundant proteins with greater than 30% sequence similarity. However, sequence similarity may not be enough to have diverse structures in the training set. Therefore, TM-Scores [53] have also been determined to validate if 3D structurally similar proteins exist in the training set. Among the pairs examined, only one exhibited a TM-Score slightly over 0.5. It is commonly considered that pairs with a TM-Score greater than 0.5 share the same fold, perhaps leading to a similarity in their three-dimensional structure. Nonetheless, the whole training set was preserved to avoid any bias resulting from excluding one combination with a value greater than 0.5. Therefore, in accordance with PASSer2.0, the “Huang Training set” has been selected as MEF-AlloSite’s training set.

The number of Fpocket-predicted pockets for proteins in the training set varies in quantity, ranging from 3 to 41; however, the majority of the proteins in the training set possess only a single allosteric site. The presence of more negative samples in larger proteins within the dataset guarantees an imbalanced representation of various protein sizes in the training data. Therefore, in order to address the imbalance in the training set, the PASSer2.0 algorithm has utilised random undersampling techniques to achieve a 1:5 ratio of positive to negative samples for each protein in the training set. The technique provides a high-performance model by inhibiting bias and increasing the quality of the training set. Therefore, following PASSer2.0, the undersampling technique was employed to achieve a 5:1 negative to the positive pocket ratio for a given protein in the training set.

#### Model training

In accordance with PASSer2.0, the base models in MEF-AlloSite have been trained using AutoGluon version 0.6.2 to inhibit any bias in comparison analysis. Also, the purpose of utilising AutoGluon is to ensure that performance enhancements are derived from the combination of 3D structural and amino acid information and feature selection rather than the model architecture, including deep learning techniques.

The labelling approach of PASSer2.0 [35] is used to determine whether a pocket found by Fpocket [54] is allosteric or not, depending on whether it includes one residue known to bind to allosteric modulators. A pocket is classified as 1 (positive) if it contains at least one residue that is identified as binding to allosteric modulators. Otherwise, it is classified as 0 (negative). A protein structure may thus have more than one positive label when the protein has more than one allosteric site. In addition, proteins that do not have a positive label have been eliminated using the technique outlined in PASSer2.0.

In summary, MEF-AlloSite initiates its predictive process by employing AutoGluon to train four distinct base models, designated as Model 1, 2, 3, and 4 (Fig. 3). Each of these models is trained using a specific feature set, namely Feature Set 1, 2, 3, and 4, respectively (Fig. 3). Subsequently, MEF-AlloSite harnesses its collective predictive capabilities to yield more robust and dependable predictions by averaging the prediction of base models. MEF-AlloSite not only underscores the efficacy of ensemble learning methodologies but also emphasizes the critical role of meticulous feature selection and seamless model integration in augmenting predictive performance for intricate biological phenomena such as protein allostery.

### Preparing test sets

ASBench [55] is a subset of the ASD that contains two datasets: (i) a core set with 235 different allosteric sites and (ii) a core-diversity set with 147 structurally varied allosteric sites [55]. The proteins in the core set are selected using two criteria: (i) protein complex should have the greatest number of allosteric protein-modulator interaction pairings at the protein’s allosteric site, determined by Ligplot+ [56]. (ii) If there are many complex structures with the same number of allosteric protein-modulator interactions, the complex with the lower resolution would be accepted. Also, structural alignments between any two allosteric sites in the 235 “Core set” complexes were calculated using the APoc approach [57] to eliminate structural redundancy.

In order to build a core-diversity set, all complexes in a core set were therefore divided into clusters using the PS-score (Pocket Similarity Score) [55] with a cutoff of 0.5, and complexes in clusters with only one member were immediately included in the final collection since they provide distinctive structural traits for the varied benchmarking set. Any proteins that also existed in the Huang Dataset were removed. As suggested in PASSer2.0, the proteins in the core diversity were removed if a protein in the test set did not have at least one positive label. As a final step, proteins in the test sets with a TM-score higher than 0.5 [58] in test cases or training sets were removed. Test 1 and Test 2 were created using a selection process in which chains with an allosteric binding site were specifically chosen for Test 1, while all chains in the complex were retained for Test 2. Keeping all chains on proteins makes for both a more realistic and challenging test scenario to identify the most promising model since the chain with allosteric residues has yet to be known for the real application of models.

The remaining 1365 proteins in ASD that are not members of the “Huang dataset” (Training set) or ASBench (test sets 1 and 2) constitute the third benchmark dataset. To construct the third test case, TM-Scores for each protein in the remaining protein against the protein in training and test 1 or 2 have been calculated. A protein having higher than 0.5 TM-Score in test 3 has been discarded since higher than 0.5 TM-Score can be structurally similar, which can result in bias to a model memorising the structure instead of learning. TM-score distribution is shown in Figs. 15, and 16.

Fpocket can identify nucleotide structures with pocket-like characteristics; however, it is important to note that these pockets cannot serve as allosteric sites for proteins. Therefore, identified pockets that only contained nucleotides were removed from all proteins in all the benchmarks. After the preprocessing steps discussed above, Table 2 shows the exact number of samples, including pocket numbers in all three test cases.

**Table 2:** The number of samples in datasets, Huang dataset (Training), ASBench with chain selection (Test 1), ASBench (Test 2), and the remaining proteins in ADS (Test 3)

Dataset	Proteins	Pockets	Allosteric sites	Allosteric site ratio (%)	Chain selection
Huang training data	90	2207	137	6.210	Yes
Test 1	56	1510	87	5.762	Yes
Test 2	56	2471	88	3.561	No
Test 3	122	6384	202	3.164	No

The third benchmark dataset comprises the proteins in ASD that are not included in the training or Tests 1 and 2. The chain selection process resulted in the allosteric site ratio for Test 1 being the most elevated among the various test instances

### Comparison with State-Of-The-Art methods

Structure-based drug discovery begins with identifying and characterising drug-binding sites [51]. The technologies that now exist include Molecular Dynamics, Network-Based and Deep Learning approaches for the identification of allosteric binding sites [15, 17, 59]. While the application of molecular dynamics has identified allosteric binding sites, it is important to acknowledge that molecular dynamics simulations present notable computational obstacles and often encounter limitations in terms of duration, resulting in insufficient sampling of the conformational space. Therefore, the prevailing problem of inadequate conformational sampling requires future efforts in algorithmic development and hardware engineering. Furthermore, allosteric regulation has been well recognised as a prevalent attribute of protein networks, and its underlying mechanisms can be understood by examining residue interaction networks. Within this particular framework, the act of an effector molecule binding initiates a sequence of interrelated fluctuations that spread throughout the network, ultimately resulting in functional reactions at remote locations. Complicated deep learning models are promising to determine orthosteric, allosteric and cryptic binding sites, such as DeepPocket [60], GraphSite [61], and PocketAnchor [62]. However, the inherent complexity of deep learning models reduces prediction interpretability [63]. On the other hand, simpler models and Fpocket features are critical to understanding complex protein allostery [1]. Due to protein allostery complexity, the locations of allosteric sites for most drug targets remain unknown [51]. Therefore, fundamental, simplest and diverse processing approaches (such as AlloPred [39]) were constructed after determining pockets to comprehend the complexity of allostery.

AlloPred [39] employs a Support Vector Machine (SVM) algorithm to establish a machine learning model using the identical attributes produced by Fpocket. In contrast, the PASSer2.0 framework incorporates AutoGluon as the underlying model instead of utilising SVM. AutoGluon is a software library designed to streamline and automate the machine learning process, specifically focusing on automating the tasks associated with Automated Machine Learning (AutoML). The tool aids in the instruction and execution of machine learning models, specifically targeting those lacking prior expertise in the domain. AutoGluon functions by automating several essential tasks, namely data preprocessing, model selection, and model training. Also, the first version of PASSer outperformed AlloPred [39], specified to identify allosteric binding sites using Fpocket. Based on the evidence mentioned above, it is plausible to assert that AlloPred has no potential to exhibit comparable performance to our model. Therefore, AlloPred was not involved in the comparison analysis of the study.

The identification of protein allosteric binding site consists of two main steps: (i) identification of cavities by using cavity detection tools, and then (ii) order of cavities to find allosteric ones. Therefore, PASSer [4], PASSer2.0 [35], and PASSerRank [36] use Fpocket to determine cavities and then use their models to select an allosteric binding site. As mentioned previously, the PASSer2.0 framework employs AutoGluon, whereas PASSerRank utilises LGBMRanker [64]. LGBMRanker is an algorithm based on a gradient boosting machine (GBM) that has been specifically developed for the purpose of rating assignments. The algorithm LightGBM is built upon the widely used GBM classification and regression method. According to the source cited [64], it provides enhanced precision, efficiency, scalability, and user-friendliness. Furthermore, the classification of allosteric sites offers a significant advantage in the categorisation of pockets by enabling the determination of a threshold for distinguishing various 3D structures and protein sequences. Thus, using LGBMRanker by PASSerRank improves the ranking efficacy of the allosteric binding site.

The other allosteric binding site identification programs use different cavity detection tools instead of Fpocket. Using another cavity detection tool results in different pocket numbers, sizes, and labels. Comparing such programs can be deceptive; therefore, only tools that use Fpocket as a main cavity detector, such as PASSer2.0 and PASSerRank, have been considered for comparison. Consequently, since the use of Fpocket and its parameters are identical for programs, the performance enhancement is exclusively the result of feature selection and the integration of 3D structural information with amino acid knowledge.

MEF-AlloSite has been developed to use multimodel feature selection and compared with the ensemble feature selection model. Following feature set selection, the features were used as a single feature set to train the ensemble feature model. AutoGluon uses more than one model to build an n-layer multi-stacking ensemble model by weighting base models. In this study, a comparison was made between Multimodel Ensemble Feature selection and ensemble feature selection.

Overall, this study utilised the MEF-AlloSite approach to conduct a series of five comparative studies. (i) The methods PASSer2.0 and PASSerRank, which are now at the cutting edge of the field, have been utilised to validate the superior performance of MEF-AlloSite compared to other state-of-the-art methods. (ii) The paper examines the notion of ensemble feature selection, called “Ensemble features,” and compares it with Multimodel Ensemble Feature selection in the MEF-AlloSite framework. (iii) An ablation study is undertaken to establish that MEF-AlloSite requires each component for improved performance. (iv) The compared model, called “Entire Features”, is trained to utilise the entire feature set in order to assess the influence of feature selection on performance. (v) An MEF-AlloSite has been compared with its components, including Model 1, 2, 3 and 4.

### Performance evaluation metrics and statistical tools

Several measurements known as performance metrics or evaluation metrics are used to assess models’ ranking and classification performance using average precision, ROC AUC, and F1 scores. The Student’s T-test and Cohen’s D value were calculated to validate the improvement of MEF-AlloSite.

Average precision score (AP): The weighted mean of precision values at each threshold is used to determine average precision; the weight represents the expected precision value for a given recall score.

ROC AUC score: The area under the receiver-operating characteristic curve (ROC AUC) score indicates the effectiveness of a model. The model performs better at separating the positive and negative classes the higher the AUC. An AUC value of 0.5 represents purely random predictions.

F1 score at top-n threshold: The F1 score combines the accuracy and recall measures into a single rating. Also, the F1 score has been intended to perform effectively with unbalanced data. The ordered F1 score, such as the F1 score at top-n, focuses on the performance of the model’s top predictions. N represent the number of top predictions accepted as “True” predictions to calculate the F1 score.

Recall at top-n threshold: Recall is a measure of how many relevant items are retrieved by a system. It is calculated as the ratio of the number of relevant items retrieved to the total number of relevant items.

Precision at top-n threshold: Precision is a measure of how many relevant items are retrieved by a system, divided by the total number of items retrieved. It is calculated as the ratio of the number of true positives to the sum of the true positives and false positives. The top-n threshold has been used to calculate the precision of models based on a ranking threshold. The proportion among top-n positions: The proportion among the top-n positions refers to the ratio of founding allosteric sites that are located inside those locations.

The confidence interval of mean and median: A confidence interval is a range of values around a sample estimate, such as a mean or median, likely to contain the true population parameter. Using a 95% confidence interval signifies that the resulting confidence intervals would include the true population mean and median.

Student’s t-test: The one-sided t-test is often utilized in experimental and observational studies to compare the means of two groups or to determine whether a sample mean significantly differs from a known value in a specific direction. This type of test is particularly useful when the research hypothesis predicts that one group will have a higher (or lower) mean than the other, allowing for a focused assessment of directional differences.

Cohen’s D: Cohen’s D is an effect size measurement that quantifies the ratio of the difference of means in two groups to the pooled standard deviation of those groups. Statistical analyses frequently employ it to assess the practical significance of a difference between two groups or conditions. There are three main effect sizes based on Cohen’s D: (i) 0.2 or less is considered a small effect size, (ii) 0.5 is considered a medium effect size, which proves significant improvement, and (iii) 0.8 or higher is considered a large effect size.

## Results and discussion

Evaluation of MEF-AlloSite is divided into three primary components: (i) A comparison analysis demonstrates that MEF-AlloSite performed better than the state-of-the-art approaches PASSer2.0 and PASSerRank. (ii) The performance of MEF-AlloSite can be analysed to gain insight into its mechanism and provide valuable information for future studies on identifying allosteric binding sites. (iii) At last, a case study demonstrating the practical use of MEF-AlloSite.

### Comparison analysis

The validation and comparison of MEF-AlloSite with PASSer2.0 and PASSerRank were conducted using two performance metrics, namely (i) Ranking Performance Comparison with Alternative Approaches and (ii) Classification Performance Comparison with Alternative Approaches.

#### Ranking performance comparison with alternative approaches

The model performance evaluation has been conducted on three distinct test sets, test 1, 2 and 3. The sole distinction between tests 1 and 2 lies in the keeping of all protein chains within the complex. Additionally, the models have undergone evaluation on test 3, which is considered as a supporting benchmark by having the highest number of proteins.

**Fig. 4:**
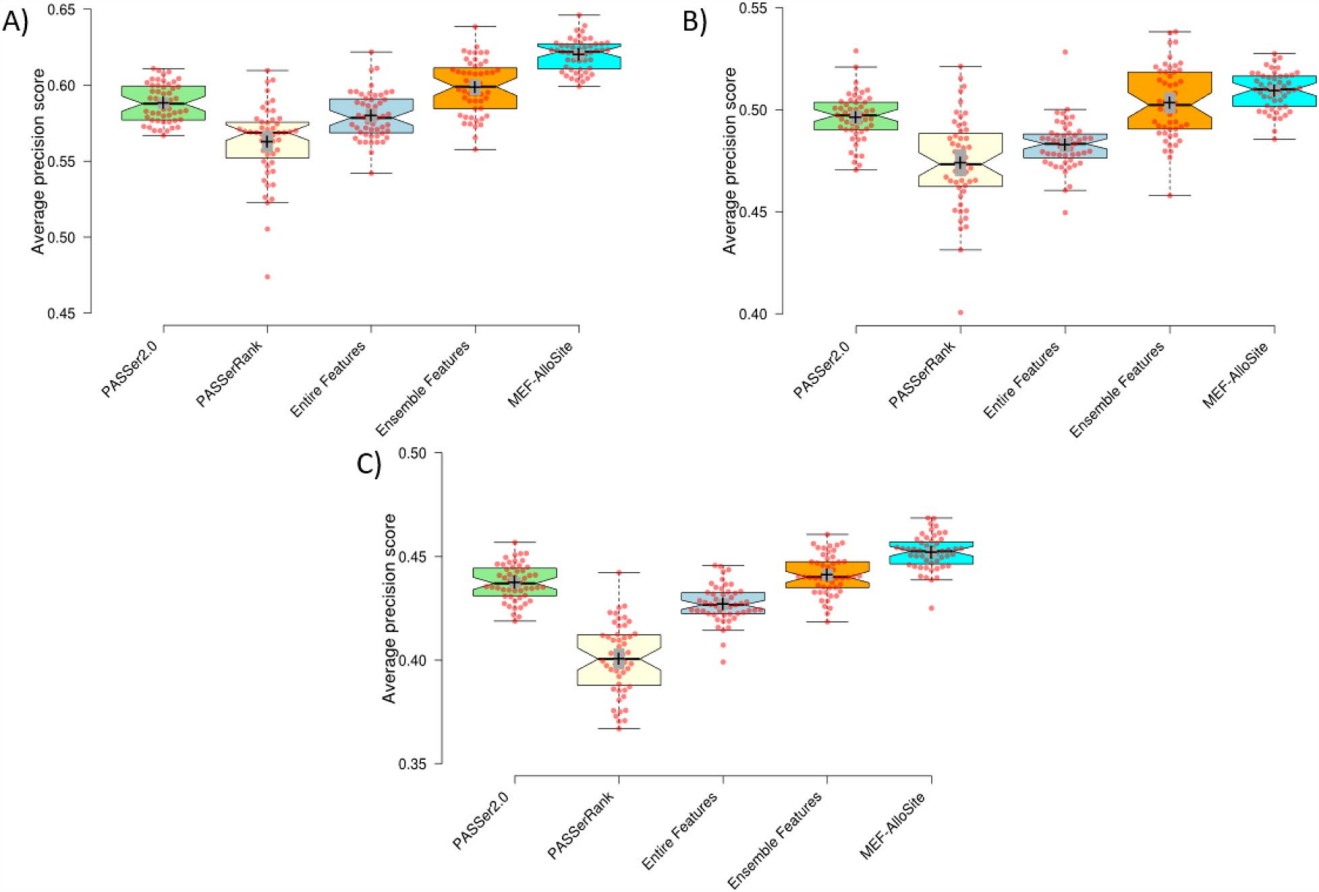
The box plots summarise the ranking performance of four models, using Average precision and ROC AUC score across 51 repeats with different splits of the training set. Box plots were created to visually show the average precision scores for Test 1, Test 2, and Test 3, denoted as **A**, **B**, and **C**, respectively. The remaining model. In this comparative analysis, two state-of-art models were evaluated, namely PASSer2.0 with a green colour scheme, PASSerRAnk, a light yellow colour. The Entire Features model has a light cyan colour scheme trained by 9460 features. The model performance effectively illustrates the impact of feature selection on performance. The Ensemble Features model with an orange colour scheme shows the performance of aggregated selected features from different feature models (Fig. 3). The MEF-AlloSite, which employs a light blue colour scheme, is utilised to compare four distinct models that have been previously mentioned. Finally, the 95% confidence intervals for model means and medians are demonstrated using plus and notches. The red dots in the visual representation correspond to the average value inside each of the 51 distinct intervals

**Fig. 5:**
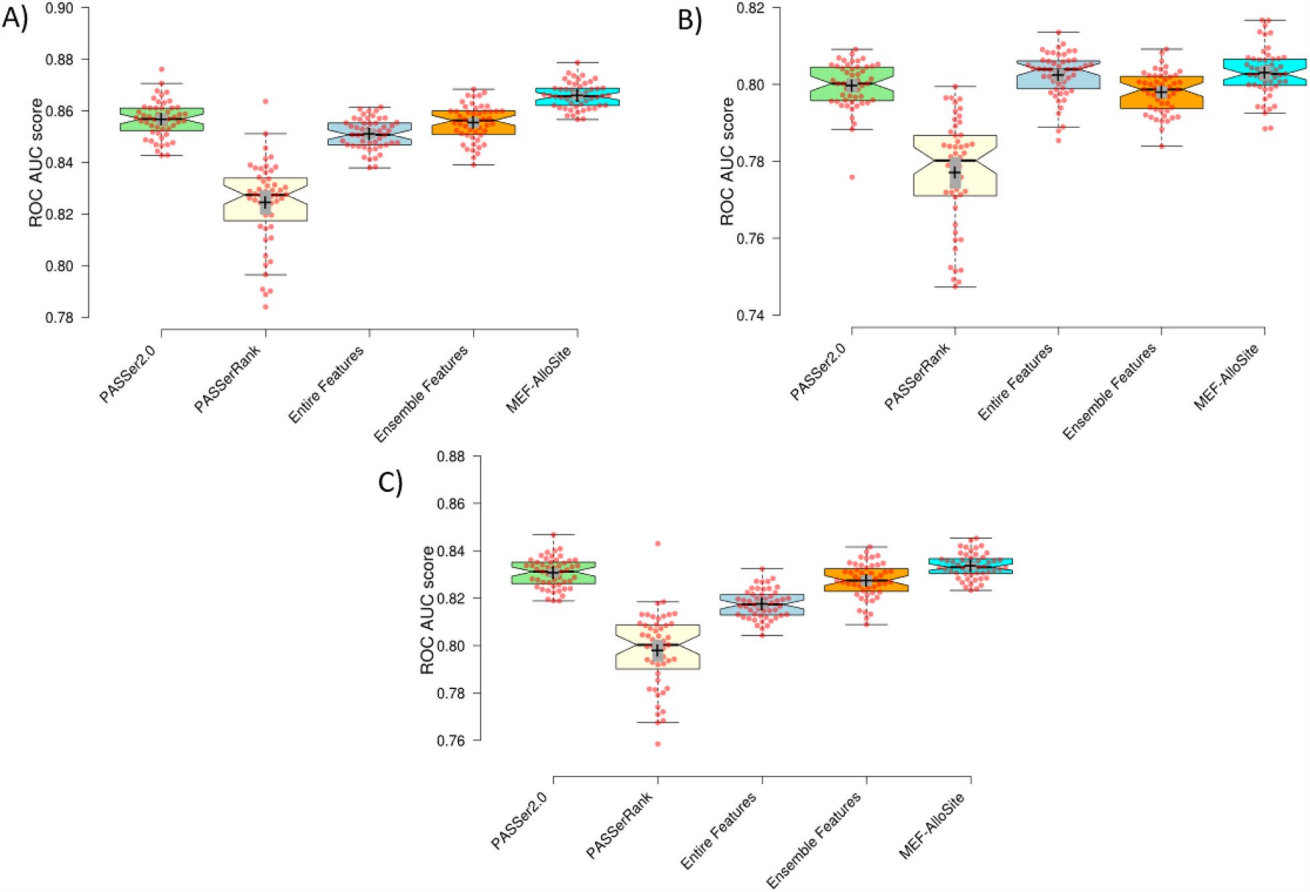
The box plots provide a summary of the ranking performance of four models. This is done by using the Average Precision and ROC AUC score. The summary is based on 51 repetitions using various divisions of the training data. Box plots were generated to graphically represent the ROC AUC scores for Test 1, Test 2, and Test 3, labelled as **A**, **B**, and **C**, respectively. This comparison investigation examined two advanced models, namely PASSer2.0 with a green colour scheme and PASSerRAnk with a light yellow tint. The Entire Features model is trained using 9460 features and has a light cyan colour scheme. The model’s performance successfully demonstrates the influence of feature selection on its performance. The Ensemble Features model, depicted in Fig. 2, displays the performance of combined selected features from various feature models. The model is presented with an orange colour scheme. The MEF-AlloSite, characterised by a light blue colour scheme, is employed to compare four specific models that have been previously stated. The 95% confidence intervals for model means and medians are illustrated using plus and notches. The crimson dots in the graphical depiction correspond to the mean value inside each of the 51 unique intervals

Figure 4 and 5 illustrates the utilisation of two established metrics, namely Average Precision and ROC AUC score, to facilitate comparative analysis across four models on three test cases. The complete set of features (9460) was utilised to train the Entire Features Model (shown in light cyan) depicted in Fig. 4 to illustrate the model’s performance without any feature selection. The results indicate that MEF-AlloSite has superior average precision compared to the entire features model, as evidenced by its higher means (+) and mean (notches) throughout the three test scenarios. The MEF-AlloSite model achieved accuracy scores of 0.620, 0.509, and 0.452 on Tests 1, 2, and 3, respectively. In comparison, the Entire Features model had accuracy scores of 0.580, 0.482, and 0.427. The data shown in the figures suggests that using a feature selection strategy holds promise in enhancing the accuracy and effectiveness of identifying allosteric binding sites. Figure 5 A, B and C were generated by utilising the distribution of ROC AUC scores from 51 distinct splits to provide evidence in favour of the feature selection. The results indicate that multimodel feature selection exhibits notably higher means and medians in two out of the three test situations. The ROC AUC scores of the feature selection model did not show significant improvement in test 3. In test 3, the MEF-Allosite feature had a mean ROC AUC score of 0.803, whereas the model using the whole feature set scored 0.798.

MEF-AlloSite employs a multimodel feature selection instead of an ensemble feature selection. The difference between them is that they use different approaches to combine features. Ensemble feature selection aggregates feature sets and then trains the final model, while multimodel feature selection uses each feature set to train base models and then aggregates the models (Fig. 1). Therefore, MEF-AlloSite has been compared with the ensemble feature selection model (orange in Fig. 4). In order to conduct a comparative analysis of the two feature selection approaches, two well-established measures, namely Average Precision and ROC AUC scores, were employed across three distinct test sets. In each of the three test instances, multimodel feature selection consistently yields superior Average Precision and ROC AUC scores (Fig. 4 and 5). For instance, the MEF-AlloSite demonstrates an average precision of 0.620 on Test 1, but the ensemble feature model achieves a lower value of 0.599. Furthermore, it was observed that MEF-AlloSite exhibited significantly higher mean values and wider confidence intervals (shown by notches) for each measure in all three test situations. Based on the results above, it can be concluded that multimodel feature selection outperforms ensemble feature selection.

According to the data presented in Fig. 4, it can be observed that PASSerRank exhibited the lowest average precision when evaluated on all three test sets. The average precision values for tests 1, 2, and 3 were recorded as 0.561, 0.476, and 0.398, respectively. Both MEF-AlloSite and PASSer2.0 demonstrated superior performance compared to PASSerRank, as seen by higher average precision scores across three separate test sets. The aforementioned pattern has been noted in three distinct experimental scenarios, wherein the evaluation of Receiver Operating Characteristic (ROC) Area Under the Curve (AUC) scores has been conducted throughout a total of 51 iterations. The data suggests that the utilisation of AutoGluon in PASSer2.0 yields superior results compared to the implementation of LGBMRanker in PASSerRank. It was anticipated that AutoGluon would exhibit comparable performance to LGBMRanker, as AutoGluon is an automated machine-learning platform with the ability to select and train a wide array of machine-learning models independently. AutoGluon possesses the capacity to explore a wider range of models and hyperparameters than an individual can feasibly accomplish manually for LGBMRanker. The heightened capacity for exploration has the promise of enhancing performance in specific situations. The automatic data preprocessing duties of AutoGluon may have contributed to the improvement in performance.

The comparison model, referred to as PASSer2.0, is depicted in light green in Fig. 4 of the publication. MEF-AlloSite exhibits a notable enhancement in average precision across three distinct test situations. In the first test, MEF-AlloSite achieved an average precision of 0.62, while PASSer2.0 obtained a value of 0.588. Furthermore, the discrepancy between MEF-AlloSite and PASSer2.0 was approximately 0.014 between tests 2 and 3. While the ROC AUC distribution for tests 2 and 3 suggests that the improvement may not be statistically significant, Fig. 5A demonstrates a noticeable distinction between the two models on test 1. This evident separation implies a considerable improvement on test 1. Statistical methods were employed to validate and analyze the results derived from the box plot (Fig. 4 and 5), including Student’s T-test and Cohen’s D value.

The box plots in Fig. 4 and 5 indicated that MEF-AlloSite has superior performance compared to PASSer2.0, Entire Feature Set, and Ensemble Feature Selection Models. Table 3 validates the observed gaps between the box plots depicted in Fig. 4 and 5, indicating a statistically significant performance improvement. A Cohen’s D value of more than 0.5 significantly impacts the statistic, as evidenced by nearly all comparisons except for one in Table 3. The Cohen’s D values in Table 3 range from 0.420 to 3.058 across three test cases against three models, signifying statistically significant enhancements. Furthermore, a Cohen’s D value greater than 0.8 indicates a larger effect on performance. Out of the 18 comparisons made (derived from 2 metrics, 3 cases, and 3 comparison models), it is seen that 14 of them exhibit Cohen’s D values that surpass 0.8, representing a large effect size.

**Table 3:** The summary of the comparison of models on Tests 1, 2 and 3

Test cases	Statistical method	Average precision				ROC AUC score			
		PASSer2.0	PASSerRank	Entire features	Ensemble features	PASSer2.0	PASSerRank	Entire features	Ensemble features
Test 1	p-value	3.43E−25	1.23E−26	3.88E−27	5.42E−11	1.16E−11	8.40E−29	4.90E−25	4.61E−14
	Cohen’s D	2.759	3.493	3.058	1.468	1.512	4.110	2.741	1.739
	Statistic	13.934	17.638	15.442	7.411	7.635	20.754	13.841	8.783
Test 2	p-value	6.29E−09	1.26E−15	4.63E−22	1.86E−02	6.14E−03	8.83E−23	5.15E−26	2.15E−06
	Cohen’s D	1.234	2.033	2.478	0.420	0.505	3.018	2.827	0.969
	Statistic	6.231	10.264	12.512	2.120	2.552	15.242	14.278	4.892
Test 3	p-value	5.09E−14	1.19E−30	6.83E−27	1.00E−08	3.95E−03	8.75E−19	3.06E−01	2.01E−05
	Cohen’s D	1.709	3.838	2.903	1.211	0.537	2.357	0.101	0.852
	Statistic	8.628	19.379	14.657	6.113	2.711	11.903	0.510	4.305

Two different ranking metrics evaluate the performance of models: average precision and ROC AUC score. Our method, MEF-AlloSite, compared with PASSer2.0, Entire feature set and Ensemble selection. The Entire Feature Set Model represents model performance that does not use any feature selection approach. The Ensemble selection method pertains to the use of AutoGluon’s n-layer stacking ensemble model and ensemble feature selection techniques. Calculated p-values were used to determine the most promising feature set. Cohen’s D values have been calculated to investigate the effect size of improvement

Another statistical tool employed for comparative analysis is the Student’s t-test. The p-values ($< 0.05$) suggest a statistically significant improvement for MEF-AlloSite. The p-values for all comparisons have been presented in Table 3. All p-values, except for the comparison of ROC AUC score performance between MEF-AlloSite and the Ensemble Feature selection model on Test 3, are observed to be significantly lower than 0.05. Therefore, the comparative analysis reveals that MEF-AlloSite has superior overall ranking performance in comparison to PASSer2.0, PASSerRank and other models, as evidenced by the data presented in Fig. 4 and 5 and Table 3.

#### Classification performance comparison with alternative approaches

The performance evaluation of the alternative model focuses on its categorisation capability. The assessment of classification performance aids in determining whether a given cavity is allosteric or not. Consequently, F1 at top 1, precision at top 1, and recall at top 1 metrics were employed to assess the efficacy of the models.

Protein architectures can exhibit significant variations, and the cavities identified by Fpocket display distinct ternary structures. At times, employing a higher threshold can yield more favourable outcomes, while alternatively, utilising a lower threshold can yield more favourable outcomes. Therefore, optimisation of the threshold (0.5) can be problematic for most of proteins. Consequently, a ranking-based approach utilising thresholds (namely, the top N predictions) was employed to compute categorisation metrics (Fig. 5).

**Fig. 6:**
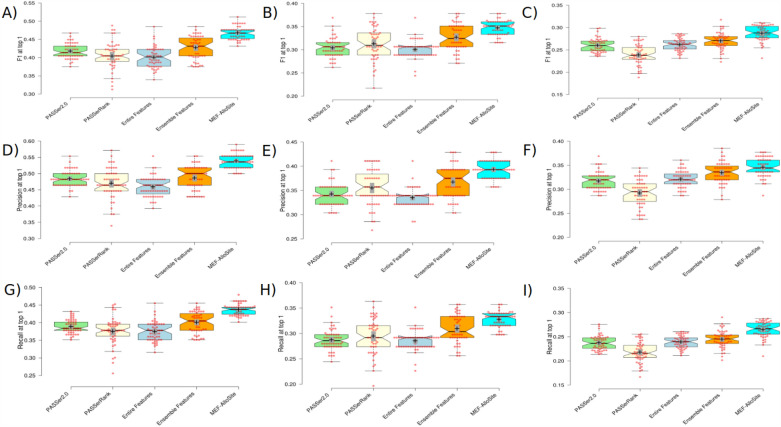
The summary of the classification performance for comparative models. The box plots for the top-1 threshold display the values of F1, Precision, and Recall. **A**, **B**, and **C** represent the distribution of F1 scores in tests 1, 2, and 3, respectively. Furthermore, **D**, **E**, and **F** exhibit precision for tests 1, 2, and 3. The final row, **G**, **H**, and **I**, represent the Recall performance for tests 1, 2, and 3 on 51 repetitions, utilising different divisions of the training data. This comparison investigation involved the evaluation of two advanced models, namely PASSer2.0 with a green colour scheme and PASSerRAnk with a bright yellow colour scheme. The model utilised in this study is the Entire Features model, which was trained using a light cyan colour scheme and a total of 9460 features. The model’s performance effectively demonstrates the influence of feature selection on its overall performance. The performance of aggregated selected features from various feature models is depicted in Fig. 3 using the Ensemble Features model, which employs an orange colour scheme. The MEF-AlloSite, characterised by its utilisation of a light blue colour scheme, serves the objective of conducting a comparative analysis among the four aforementioned models. The model’s classification performance has been evaluated by employing three classification metrics: F1, Precision, and Recall at top 1. The true prediction label has been assigned to the top 1 prediction of each model to generate classification metrics. The 95% confidence intervals for model means (+) and medians (notches) are illustrated utilising plus and notches. The red dots depicted in the visual depiction correspond to the average value included within each of the 51 unique intervals

The Entire Feature Model (highlighted in cyan) has been employed to assess the performance of a model without any feature selection. In test 1, Fig. 6A, D, and G correspond to the evaluation metrics F1 score, Precision, and recall score, respectively. Figure 6A, D, and G demonstrate that the Entire Feature Model exhibited the lowest F1, precision, and recall scores, respectively. The findings shown in Fig. 6B, C, E, F, H, and I reveal that the Entire Feature Model and PASSerRank classification performance were the lowest on tests 2 and 3. In contrast, the results obtained by MEF-AlloSite demonstrate noticeably higher mean values (+) and median (notches) intervals than those obtained from the Entire Features Model. This observation suggests that our feature selection methodology positively impacts the overall classification performance.

The MEF-AlloSite model has higher means and non-overlapping medians on the F1, precision, and recall box plots compared to the Ensemble Feature selection model (orange colour, Fig. 6). Hence, it can be observed from Fig. 6 that MEF-AlloSite exhibits superior performance compared to Ensemble Feature Selection Model.

The assessment of model ranking is frequently conducted by utilising average precision and ROC AUC metrics. Based on the analysis of average precision and ROC AUC score distribution, it can be observed that PASSerRank (light yellow, Fig. 6) demonstrated the least satisfactory performance when compared to the other three approaches. Also, MEF-AlloSite exhibited the most superior classification performance among the five models, which included PASSer and PASSerRank (Fig. 6).

As for the comparison with PASSer2.0 (green, Fig. 6), as a published study, the box plots in Fig. 6 demonstrate that MEF-Allosite provides a clear performance improvement against PASSer2.0. MEF-AlloSite improves F1 scores by 5.000%, 4.300% and 2.699% on Tests 1, 2 and 3. Also, the precision and recall score support that MEF-AlloSite has better classification performance than PASSer2.0. To statistically validate the deductions from the box plots presented in Fig. 6, the Student’s t-test was employed, and the Cohen’s D value was calculated.

Table 4 presents the statistical data for conducting a comparative analysis of MEF-AlloSite with three different models, namely PASSer2.0, Entire Feature Set, and Ensemble Feature Selection. In order to conduct a comparative analysis of these models, three pre-defined test cases were employed. The statistical significance of our deductions from box plots, based on mean and median intervals, is supported by p-values ($< 0.05$). Specifically, the MEF-AlloSite technique demonstrates a higher degree of accuracy in identifying the allosteric binding site than the other three methods, as evidenced by its top-ranked prediction.

**Table 4:** The analysis of performance comparison in classification using F1 score

Test cases	Statistical method	F1 at 1			
		PASSer2.0	PASSerRank	Entire features	Ensemble eeatures
Test 1	p-value	2.08E−24	1.01E−21	1.09E−24	2.17E−12
	Cohen’s D	2.677	2.732	2.868	1.618
	Statistic	13.520	13.794	14.485	8.170
Test 2	p-value	1.86E−20	7.06E−09	7.26E−24	1.69E−05
	Cohen’s D	2.333	1.271	2.637	0.872
	Statistic	11.780	6.420	13.318	4.404
Test 3	p-value	1.24E−13	9.11E−22	2.62E−13	1.79E−06
	Cohen’s D	1.674	2.460	1.653	0.972
	Statistic	8.454	12.421	8.345	4.910

The performance of MEF-AlloSite has been evaluated and compared with that of PASSer2.0, the Entire Feature Set model, and the Ensemble selection model. The initial forecast of each model is designated as a “True” prediction, and afterwards, the F1 score is computed for each model. The statistical analysis involved utilising the F1 scores distribution from 51 distinct splits, applying the Student’s T-test and calculating Cohen’s D value

Cohen’s d values provide a measure of effect size for improvement. When the value of Cohen’s d exceeds 0.5, it indicates a medium effect size and statistically significant improvement. An effect size greater than 0.8 signifies a bigger magnitude of influence, indicating a distinction between groups or conditions. The evident separation does not necessitate using a statistical test for validation. The MEF-AlloSite model exhibits a Cohen’s D value over 0.8 in three separate test situations. This observation strongly indicates the significant potential of our model in accurately identifying allosteric binding sites as the top-ranked prediction.

Table 5 demonstrates that precision and recall scores are based on top-ranked prediction to understand F1 score improvement (Table 4) and validate MEF-AlloSite performance. A greater precision score signifies that the positive predictions made by the model are more dependable and exhibit a reduced occurrence of false positives. The p-value ($< 0.05$) and Cohen’s D value ($> 0.8$) strongly suggest that MEF-AlloSite outperforms all other benchmark algorithms.

**Table 5:** The summary of precision and recall performances for comparison analysis

Test cases	Statistical method	Precision at Top 1				Recall at Top 1			
		PASSer2.0	PASSerRank	Entire features	Ensemble features	PASSer2.0	PASSerRank	Entire features	Ensemble features
Test 1	p-value	8.66E−22	1.37E−22	1.95E−26	7.08E−14	7.99E−25	3.76E−21	6.25E−24	1.20E−11
	Cohen’s D	2.419	2.757	2.974	1.757	2.727	2.683	2.805	1.543
	statistic	12.217	13.923	15.017	8.871	13.771	13.548	14.164	7.793
Test 2	p-value	1.14E−21	2.63E−09	1.20E−28	2.30E−06	1.21E−19	1.17E−08	1.25E−21	4.27E−05
	Cohen’s D	2.434	1.310	3.073	0.975	2.260	1.249	2.437	0.821
	statistic	12.293	6.614	15.520	4.923	11.411	6.309	12.304	4.148
Test 3	p-value	9.67E−12	2.18E−19	5.10E−10	3.68E−03	2.07E−14	3.32E−22	2.93E−14	1.84E−08
	Cohen’s D	1.499	2.246	1.338	0.542	1.745	2.495	1.745	1.182
	statistic	7.570	11.342	6.756	2.737	8.813	12.601	8.810	5.970

The evaluation and comparison of MEF-AlloSite’s performance have been conducted in relation to PASSer2.0, the Entire Feature Set model, and the Ensemble selection model. The initial predictions made by the models are designated as “True” in order to calculate precision and recall. The statistical study entailed the usage of the distribution of F1 scores obtained from 51 separate splits. Additionally, the analysis comprised the application of the Student’s T-test and the computation of Cohen’s D value

### MEF-AlloSite performance analysis

Gaining insight into the enhanced efficacy of MEF-AlloSite can aid in comprehending the phenomenon of allostery in target proteins. Thus, there are three primary inquiries: (i) Ablation analysis, (ii) Assessment of ensemble models, and (iii) Performance analysis of multimodel feature selection.

#### Ablation analysis

Individual components of MEF-AlloSite have been systematically eliminated in order to gain a deeper understanding of the model’s functioning and elucidate the interplay between protein allostery (Table 6).

**Table 6:** The summary of Ablation analysis on three test cases

Ensemble model without a component	Statitical method	Test 1		Test 2		Test 3	
		Average precision	ROC AUC score	Average precision	ROC AUC score	Average precision	ROC AUC score
No Model 1	p-value	2.72E−01	1.91E−01	2.49E−01	9.24E−01	8.52E−07	3.29E−04
	Cohen’s D	0.120	0.174	0.135	− 0.285	1.008	0.697
No Model 2	p-value	3.22E−05	0.188	1.25E−02	0.301	5.26E−01	0.521
	Cohen’s D	0.826	0.176	0.451	0.103	− 0.013	− 0.010
No Model 3	p-value	3.94E−02	8.31E−04	2.75E−01	5.32E−04	4.36E−01	5.15E−01
	Cohen’s D	0.352	0.640	0.119	0.668	0.032	− 0.008
No Model 4	p-value	7.03E−01	6.85E−01	4.27E−02	6.97E−02	1.42E−03	1.23E−01
	Cohen’s D	− 0.106	− 0.096	0.344	0.295	0.606	0.231

MEF-AlloSite contains four models, so each model has been discarded one by one from the pipeline to investigate its impact on performance. Constructed four ensemble models by discarding one component have been tested on three test cases, Test 1, 2 and 3. MEF-AlloSite has been compared with these four models using statistical methods for the comparison analysis. The analysis employed the Student’s T-test and involved the computation of Cohen’s D statistic. The statistical significance of the q-values ($< 0.05$) and the effect size measured by Cohen’s D value ($> 0.5$) indicate that MEF-AlloSite outperforms models with three components

Table 6 presents the results of the statistical analysis conducted to compare MEF-AlloSite with its components, using the Student’s t test and Cohen’s D value. The statistical significance of the q-values ($< 0.05$) and the effect size measured by Cohen’s D value ($> 0.5$) indicate that MEF-AlloSite outperforms models with three components in Table 6. The utilisation of multimodel feature selection is motivated by its ability to achieve superior and robust performance. When examining three benchmarks, it was observed that MEF-AlloSite showed consistent performance throughout all three test conditions, with no decline in performance exceeding two measures. For instance, Feature 2 in Table 9) has been used to build Model 2. After excluding model 2 (“No model 2” in Table 6), the subsequent model comprising three models exhibited comparable Average Precision and ROC AUC scores on Test 3. Nevertheless, it is worth noting that MEF-AlloSite demonstrated a statistically significant enhancement in the average precision score for both Test 1 and Test 2. Furthermore, it was observed that the MEF-Allosite yielded a higher ROC AUC score for both test 1 and test 2. Another example may be noticed when model 4 is excluded (“No Model 4” in Table 6). The revised model, without model 4, shows competitiveness in test 1. However, the MEF-Allosite exhibited statistically significant improvements in average precision for tests 2 and 3, as indicated by p-values ($< 0.05$). Additionally, the MEF-AlloSite intervention demonstrated a rather modest impact on the ROC AUC score in Tests 2 and 3.

A comparative analysis was conducted between MEF-AlloSite and four different models, as outlined in Table 6. The evaluation was performed using two metrics, Average Precision and ROC AUC score, across three distinct test cases. Multiplying the number of models (4) by the number of metrics (2) and the number of test sets (3) resulted in a total of 24 evaluations. MEF-AlloSite showed improvement in eighteen out of twenty-four situations. Nine of eighteen have a statistical improvement, and the statistical improvement showed better performance against the ensemble model without a component across three test cases at least twice metrics across three test cases. However, the performance of MEF-AlloSite was diminished in just six out of twenty-four metrics. Overall, utilising four models in MEF-AlloSite yields superior and robust performance.

#### Ensemble Model Assessment

The MEF-AlloSite model is composed of four individual models trained using distinct feature selection methods. Subsequently, each model is assigned a linear weight to form an ensemble model. Nevertheless, the ensemble model is expected to perform better than the individual base models. Hence, MEF-AlloSite has been evaluated against baseline models across three distinct test cases, employing two evaluation metrics: average precision and ROC AUC score.

**Fig. 7:**
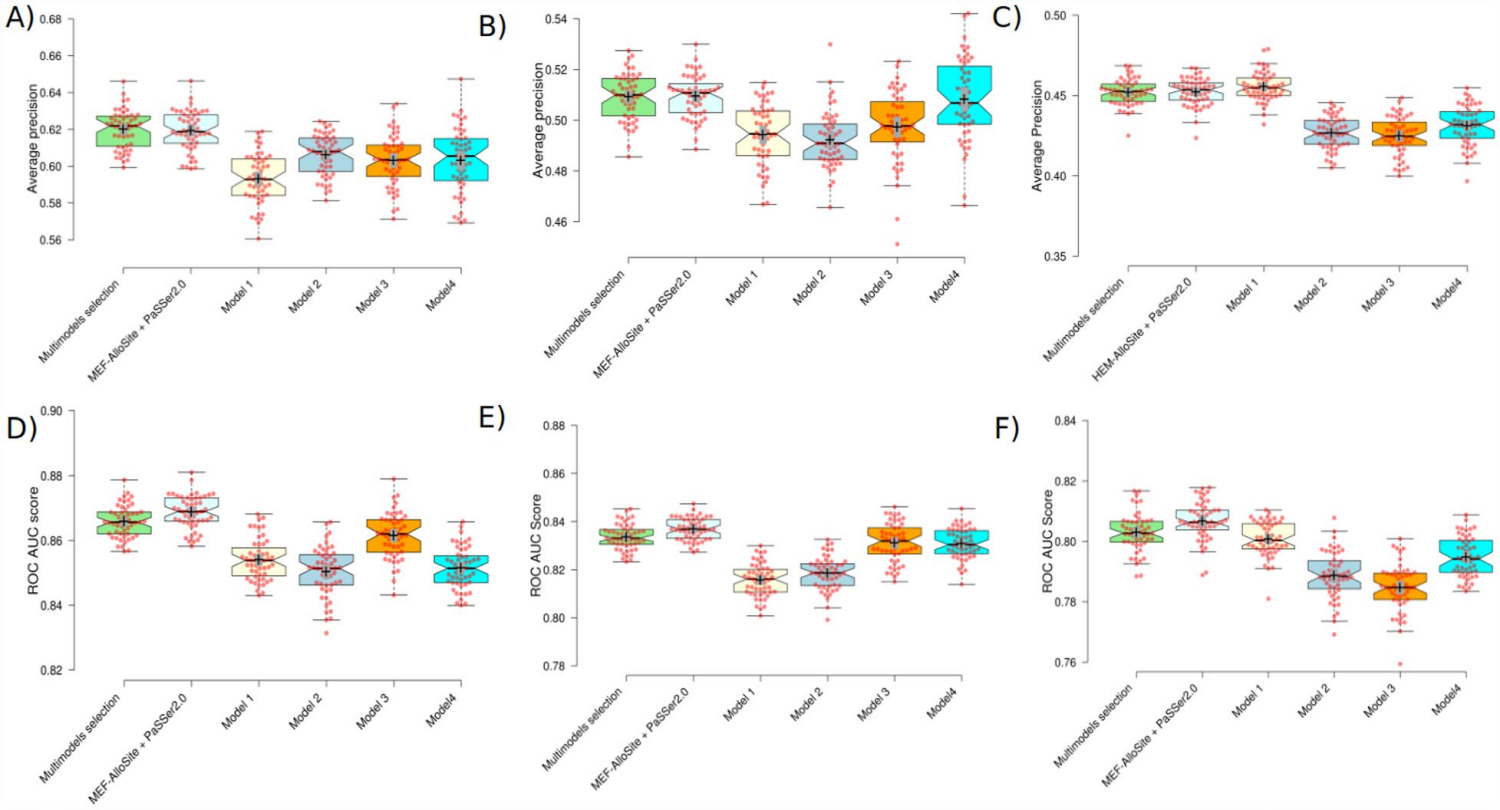
The comparison of MEF-AlloSite components using box plots. The box plots show the ranking performance of models using two metrics, average precision and ROC AUC score. **A**, **B** and **C** shows the average prediction score for Test 1, 2 and 3, respectively. Also, **D**, **E** and **F** indicate the ROC AUC score of models on Test 1, 2 and 3,  respectively. The MEF-AlloSite platform has four distinct models. In order to accurately depict model structures that incorporate more components and yield more successful outcomes, the MEF-AlloSite + PASSer2.0 model was developed. Consequently, MEF-AlloSite has been subjected to comparative analysis with five distinct models. The models used in this study are MEF-AlloSite + PASSer2.0, represented by the colour light cyan. Additionally, Model 1 is represented by light yellow, Model 2 by light blue, Model 3 by orange, and Model 4 by cyan

The results shown in Fig. 7 indicate that MEF-AlloSite exhibits superior average accuracy and ROC AUC scores in both Test 1 (Fig. 7A and D) and Test 2 (Fig. 7B and E). On the other hand, Model 1, shown by the light yellow colour in Fig. 7C and F, has a competitive performance on Test 3 despite being trained only on Feature Set 1, as indicated in Table 7. The statistical analysis reveals that the higher means (+) and medians (notches) provide compelling evidence of the superior performance of MEF-AlloSite compared to the constituent models.

**Table 7:** The summary of ensemble model performance against base models

Individual model	Statitical method	Test 1		Test 2		Test 3	
		Average precision	ROC AUC score	Average precision	ROC AUC score	Average precision	ROC AUC score
Model 1	p-value	2.59E−19	1.07E−17	1.65E−10	9.46E−27	9.79E−01	3.34E−02
	Cohen’s D	2.219	2.066	1.392	2.940	− 0.408	0.367
Model 2	p-value	1.57E−09	8.33E−21	2.54E−13	1.18E−21	5.79E−25	4.52E−17
	Cohen’s D	1.288	2.419	1.652	2.442	2.743	1.997
Model 3	p-value	2.33E−10	2.54E−04	1.43E−06	3.41E−02	2.08E−23	7.12E−23
	Cohen’s D	1.379	0.714	0.993	0.366	2.655	2.541
Model 4	p-value	3.55E−08	2.43E−22	3.53E−01	1.00E−02	2.03E−16	3.98E−09
	Cohen’s D	1.174	2.506	0.075	0.468	1.979	1.248

The MEF-AlloSite framework consists of four basic models, which are then compared with the MEF-AlloSite model. To assess the comparative performance and ranking performance, The Student’s t-test was employed, and Cohen’s D effect size was calculated. Statistical methods were employed to assess the superiority of MEF-AlloSite against individual component methods, namely Models 1, 2, 3, and 4

Table 7 displays the comparison analysis with the MEF-AlloSite component for three test cases. A p-value below 0.05 signifies that the improvement is statistically significant. Only two cases in Table 7 are greater than 0.05; therefore, MEF-AlloSite performed better than its components. Cohen’s D value is the other analysis utilised to determine the effect magnitude of improvement. Greater than 0.5 indicates a moderate effect size, a statistically sufficient improvement. In addition, Cohen’s D values greater than 0.8 indicate a sizeable effect, which is another indication of progress. Except for two Cohen’s D values in Table 7, the remaining values range from 0.366 to 2.94, indicating that MEF-AlloSite has sufficient evidence to demonstrate that it provides superior and robust performance compared to its components.

**Fig. 8:**
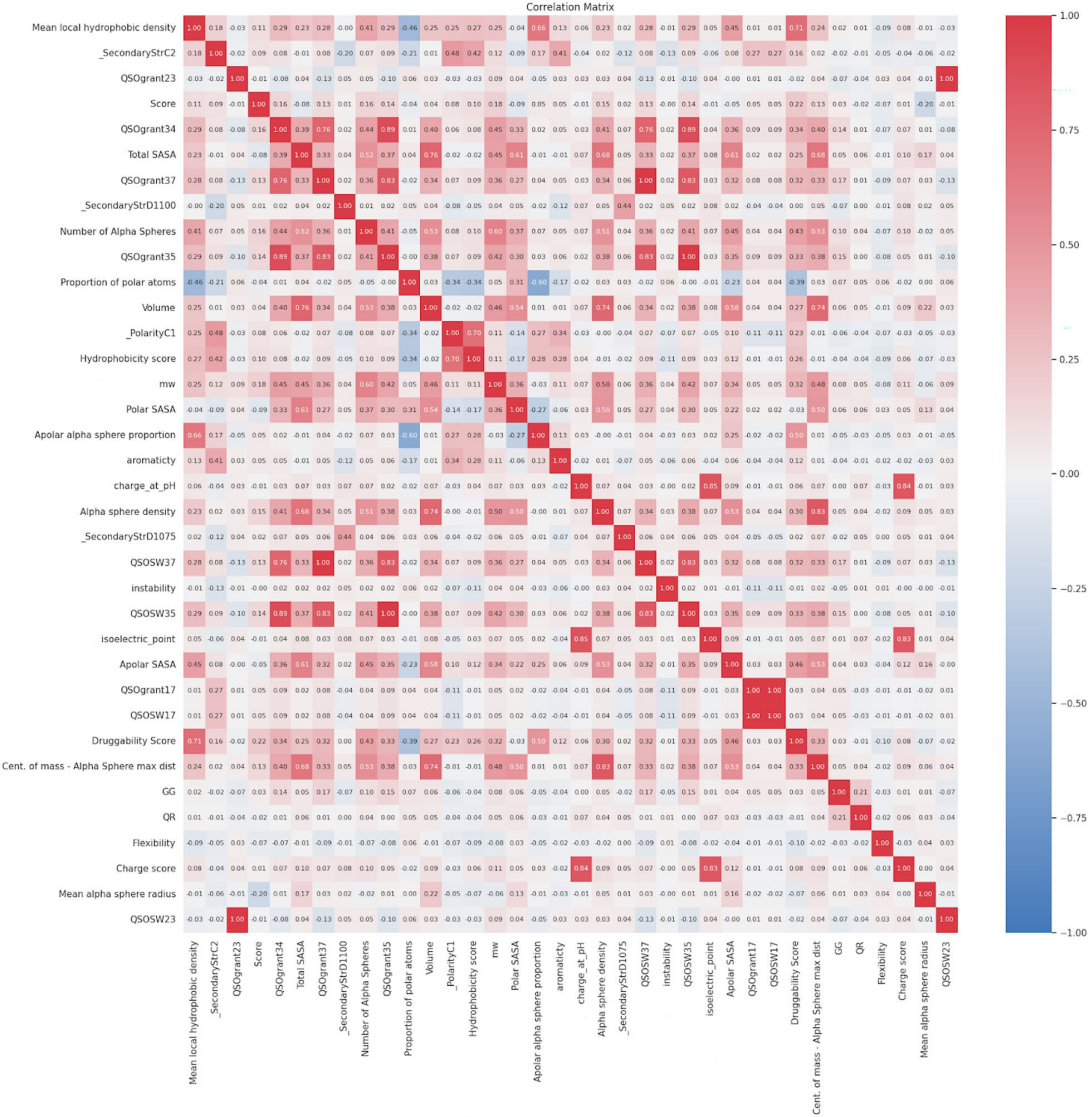
The summary of feature correlation following the outputs of aggregated feature selections, including Feature Set 1, 2, 3 and 4. Each feature shown in the correlation matrix has been detected at least once during the multimodel feature selection. The correlation coefficient is typically standardised to a range of − 1 to 1. The presence of a negative correlation in the blue region indicates a reverse correlation, whilst the positive values in the red region indicate a positive correlation between the features. White or whitish cells suggest the absence of any discernible positive or negative link between the features

MEF-AlloSite, a versatile model, can enhance the efficacy of allosteric binding sites by incorporating additional feature selection outcomes. To assess the functionality of MEF-AlloSite, PASSer2.0 was employed as a constituent of MEF-AlloSite and compared to the original structure of MEF-AlloSite. In order to assess the comparability between two distinct versions of MEF-AlloSite, statistical analysis was conducted using the Student’s T-test and Cohen’s D value, as presented in Table 8. Additionally, the performance of MEF-AlloSite can be enhanced by assigning weights to the basic models. This is because Models 3 and 4 (Fig. 8) have demonstrated greater success compared to Models 2 and 3. Thus, the weighting base model has great potential following multimodel feature selection. Additional examples include the integration of alternative models, such as ensemble feature selection, which involves picking subsets using multiple feature selection approaches and mixing them into a single base model. Furthermore, the inclusion of no feature selection models may also enhance the performance of MEF-AlloSite. These examples demonstrate the adaptability and usefulness of MEF-AlloSite.

**Table 8:** The comparison analysis statistical summary for increased component numbers of MEF-AlloSite

PASSer2.0 + MEF-AlloSite	Statitical method	Test 1		Test 2		Test 3	
		Average precision	ROC AUC Score	Average precision	ROC AUC Score	Average precision	ROC AUC Score
	p-value	6.50E−01	1.97E−03	4.44E−01	5.50E−04	4.53E−01	2.15E−03
	Cohen’s D	− 0.076	0.584	0.028	0.666	0.024	0.579

The MEF-AlloSite method employs a multimodel feature selection strategy, which consists of four distinct models. This approach has the potential to significantly enhance performance, particularly when a successful model is included in the pipeline. Hence, the integration of PASSer2.0 into the MEF-AlloSite pipeline has been undertaken to enhance the identification of allosteric binding sites. The comparative analysis has been subjected to statistical testing on three separate occasions, on tests 1, 2, and 3. The statistical analysis utilised the Student’s T-test and included the calculation of Cohen’s D value. The statistical investigation has been conducted to evaluate the superiority of PASSer2.0 + MEF-AlloSite against MEF-AlloSite

By incorporating PASSer2.0 into the MEF-AlloSite pipeline, there has been a statistically significant improvement in the ROC AUC score across three test cases (Table 8). The results presented in Table 8 indicate that MEF-AlloSite can enhance performance when provided with an informative feature set, serving as an additional model based on p-value ($< 0.05$) and a Cohen’s D value ($> 0.5$). The inclusion of even one feature set into the MEF-AlloSite pipeline leads to a significant improvement in the overall performance of predicting allosteric binding sites.

#### Multimodel Feature Selection performance analysis

The selection of features plays a crucial role in enhancing the performance of the machine learning model. The efficacy of a given feature selection method is influenced by various factors, particularly when the training data is constrained in quantity, such as in the case of the ADS data pertaining to the allosteric binding site. In order to understand the efficacy of the feature selection method, three primary areas are explored to comprehend the intricacies of feature selection in the context of protein allostery: (i) the analysis of selected features based on selection frequency, (ii) an evaluation of the importance of features in order to identify allosteric binding sites and (iii) the assessment of correlations among the selected features.

##### The analysis of selected features based on selection frequency

Certain characteristics are examined, which is crucial for comprehending the fundamental processes of protein allostery. The aim is to reveal possible relationships and determine the relevance of selected variables in clarifying allosteric processes using various selection approaches, including (i) ensemble feature selection and (ii) multimodel feature selection. Both individuals employ many feature selection approaches at the outset of their respective processes. Ensemble feature selection involves the combining of many feature sets into a unified feature set, whereas multimodel feature selection entails training individual models for each feature set and subsequently utilising them within an ensemble framework. Backward selection was utilized to optimize the feature set number in the ensemble model. Finally, four feature sets to train base models have been selected to construct MEF-AlloSite (Table 9).

**Table 9:** The comprehensive compilation of features chosen by their own feature selection methodologies

Classifier		Feature selection method	Feature	Feature important ranking	Source
Feature set 1	Random Forest Classifier	Boruta	Score	1	Fpocket
			Druggability score	2	Fpocket
			Number of Alpha spheres	3	Fpocket
			Total SASA	4	Fpocket
			Polar SASA	5	Fpocket
			Apolar SASA	6	Fpocket
			Volume	7	Fpocket
			Mean local hydrophobic density	8	Fpocket
			Apolar alpha sphere proportion	9	Fpocket
			Hydrophobicity score	10	Fpocket
			Charge score	11	Fpocket
			Proportion of polar atoms	12	Fpocket
			Alpha sphere density	13	Fpocket
			Cent. of mass - Alpha Sphere max dist	14	Fpocket
			MW	15	Biopython
			Charge_at_pH	16	Biopython
			QSOSW35	17	PyBioMed
			QSOSW37	18	PyBioMed
			QSOgrant37	19	PyBioMed
			_SecondaryStrC2	20	PyBioMed
			_PolarityC1	21	PyBioMed
Feature set 2	Gradient Boosting Classifier	Boruta	Druggability Score	1	Fpocket
			Number of Alpha Spheres	2	Fpocket
			Apolar SASA	3	Fpocket
			charge_at_pH	4	Biopython
			_SecondaryStrD1100	5	PyBioMed
Feature set 3	AdaBoosting Classifier	Model based	Score	1	Fpocket
			Number of Alpha Spheres	2	Fpocket
			Mean alpha sphere radius	3	Fpocket
			Druggability Score	4	Fpocket
			Aromaticty	5	Biopython
			Isoelectric_point	6	Biopython
Feature set 4	Gradient Boosting Classifier	Model based	Number of Alpha Spheres	1	Fpocket
			MW	2	Biopython
			Druggability Score	3	Fpocket
			QSOgrant34	4	PyBioMed
			Score	5	Fpocket
			QSOgrant23	6	PyBioMed
			Mean local hydrophobic density	7	Fpocket
			QSOgrant35	8	PyBioMed
			QSOSW17	9	PyBioMed
			QSOSW23	10	PyBioMed
			QSOgrant17	11	PyBioMed
			Volume	12	Fpocket
			QSOSW35	13	PyBioMed
			GG	14	PyBioMed
			_SecondaryStrD1075	15	PyBioMed
			QR	16	PyBioMed
			Charge_at_pH	17	Biopython
			Flexibility	18	Fpocket
			Aromaticty	19	Biopython
			Mean alpha sphere radius	20	Fpocket
			Instability	21	Biopython

The MEF-AlloSite system has four distinct models, each trained using a unique feature set, as illustrated in the table. Two of the selected features are chosen from the Boruta algorithm, while the remaining features are derived from the model-based feature selection method via a backward step-wise selection process. The multimodel feature selection approach involved the utilisation of three distinct classifiers, namely Random Forest, Gradient Boosting, and AdaBoosting Classifier, for the purpose of selecting features. Furthermore, the table presents the demonstration of feature relevance ranking and feature source

Boruta feature selection is considered a robust and effective method for feature selection in various machine learning and data analysis tasks. Therefore, the Boruta package was used to select the most informative features. Two Boruta feature sets have been selected after backward stepwise selection, in addition to two model-based feature sets. Three out of four feature selections have used different classifiers, including Random Forest, Gradient Boosting and ADABoosting Classifier (Table 9). Each feature selection method has the same feature with different orders, such as Score from Fpocket. Boruta (+ Random Forest) found the Score feature found by Fpocket (Table 9) as the most important feature to define an allosteric binding site, while it is specifically designed for orthosteric binding site identification, while Boruta (+ Gradient Boosting ) did not select Score in the final list. The model-based (+ ADABoosting) found the Score feature as the most important feature, like the Boruta (+ Random Forest) feature selection (Table 9). However, model-based (+ Gradient Boosting ) found the Score function as the fifth promising feature (Table 9).

While conducting the feature selection process, it was seen that certain features exhibited similar characteristics but were ranked differently in terms of importance. The varying ranks assigned to features highlight an important observation. There is a link between the chosen traits and protein allostery, although the strength of this association varies greatly. Throughout all feature selection methods utilised, some properties constantly appear significant but with varying rankings. This subtle differential emphasises the intricate nature of the connection between characteristics and protein allostery, indicating that some attributes may have a more significant impact on the phenomena than others.

The consistent selection of the Druggability Score and the Number of Alpha Spheres from Fpocket across all four feature selection methods underscores their potential significance in elucidating the relationship between features and protein allostery (Table 9). The Druggability Score, designed to assess the propensity of a binding site to accommodate small-molecule ligands, suggests a structural characteristic that may influence the allosteric regulation of proteins by affecting their interaction with allosteric modulators. Similarly, the Number of Alpha Spheres from Fpocket, which quantifies the surface pockets on a protein structure, may offer insights into the spatial distribution and accessibility of allosteric sites. The consistent selection of these features across four feature selections implies their relevance in capturing structural attributes that contribute to protein allostery, highlighting their potential utility in predictive modelling and mechanistic studies aimed at understanding allosteric regulation. Further analysis and validation are needed to elucidate the specific roles of these features in comprehensively modulating protein function and allosteric behaviour.

The repeated selection of both the charge_at_pH feature from Biopython and the Score feature from Fpocket across three out of four feature selection methods suggests their potential relevance in characterizing the relationship between protein allostery and structural properties (Table 9). The charge_at_pH feature calculates the overall charge of amino acids at a certain pH. The charge_at_pH feature might indicate differences in electrostatic interactions within the protein structure, which are important for allosteric communication and control. Alternatively, the Score feature in the Fpocket can indicate the druggability or potential for ligand binding in protein pockets. This feature can identify areas that might potentially function as allosteric sites or affect the binding of allosteric modulators. The repeated use of these characteristics emphasises their importance in capturing the structural and physicochemical properties that may influence the allosteric behaviour of proteins.

The inclusion of MW (molecular weight) and Aromaticity from Biopython, as well as QSOSW35 and QSOSW37 from PyBioMed, in two of the four feature selection techniques, indicates their potential importance in understanding the connection between protein allostery and molecular descriptors (Table 9). The molecular weight of a protein is a key factor that determines its size and mass. The molecular weight, in turn, has a significant impact on the protein’s structural stability and its capacity to interact with other molecules, which is necessary for allosteric control. Also, the QSOSW35 and QSOSW37 descriptors, which are linked to the distribution of charges and the hydrophobic nature of molecules, have crucial functions in the interactions between proteins and between proteins and ligands. These interactions are vital for the transmission of signals through allosteric signalling pathways. Additionally, aromaticity, which is determined by the presence of aromatic amino acids, is especially significant because aromatic residues are involved in allosteric regions and play a crucial role in facilitating conformational changes. The repeated selection of these descriptors emphasises their potential significance in comprehending the molecular foundation of protein allostery. It emphasises opportunities for more exploration into their distinct functions and processes in allosteric control.

The selection of QSOgrant37, SecondaryStrC2, QSOgrant34, QSOgrant35, QSOSW17, QSOSW23, QSOgrant17, SecondaryStrD1075, GG QR, and SecondaryStrD1100 from Pybiomed, alongside isoelectric_point from Biopython, only once in the four feature selection methods, highlights their potential relevance to the study of protein allostery (Table 9). Although their individual selection frequency may be somewhat smaller than other qualities, their inclusion implies distinct characteristics that might have significant impacts on allosteric regulation. For example, the characteristics isoelectric_point and SecondaryStrC2 can indicate the electrostatic environment and secondary structure composition of the protein, respectively. Both of these factors are known to affect allosteric behaviour. The QSO and QSOSW descriptors, which are related to the distribution of charges and hydrophobicity, provide valuable information on the physicochemical characteristics of the protein surface. This information might be relevant to understanding allosteric binding sites and conformational changes. Furthermore, characteristics such as GG, QR and SecondaryStrD1100 might potentially indicate structural motifs or sequence patterns that are involved in allosteric communication pathways. Although chosen just once, these characteristics justify more examination to clarify their precise functions and contributions to protein allostery, perhaps offering a new understanding of allosteric processes and regulatory networks.

Feature Set 4 stands out for its extensive inclusion of distinctive features compared to the other sets (Table 9), suggesting a potentially comprehensive representation of relevant characteristics. Conversely, Feature Set 1, while still substantial, harbours a slightly smaller number of features as the primary selection. Feature Sets 2 and 3, however, have fewer features, possibly reflecting a focused selection aimed at enhancing performance through feature reduction. While larger feature sets may offer a broader scope of information, they also pose challenges related to computational complexity and potential redundancy. On the other hand, smaller feature sets streamline the analysis but risk overlooking crucial aspects of the data. The varying numbers across these sets hint at the complexity of the feature selection process and the need to strike a balance between inclusivity and efficiency. The varying numbers also indicate that feature selection in allostery needs an accurate and robust approach like the multimodel feature selection technique.

Volume (Fpocket) and Total SASA (Fpocket) have a 0.76 correlation score (Fig. 8), a reasonable correlation score since a larger volume pocket can be more accessible than a smaller pocket. Also, the positive correlations between Volume (Fpocket) and Apolar alpha sphere proportion (Fpocket) and Alpha Sphere Max Dist (Fpocket) have been expected because of their direct relationship to the shape of the pocket (Fig. 8). Finding informative shape-related features for protein allostery indicates that a complex shape-matching or geometric deep-learning model can improve the allosteric binding site identification performance (Fig. 8).

##### An evaluation of the importance of features in order to identify allosteric binding sites

**Fig. 9:**
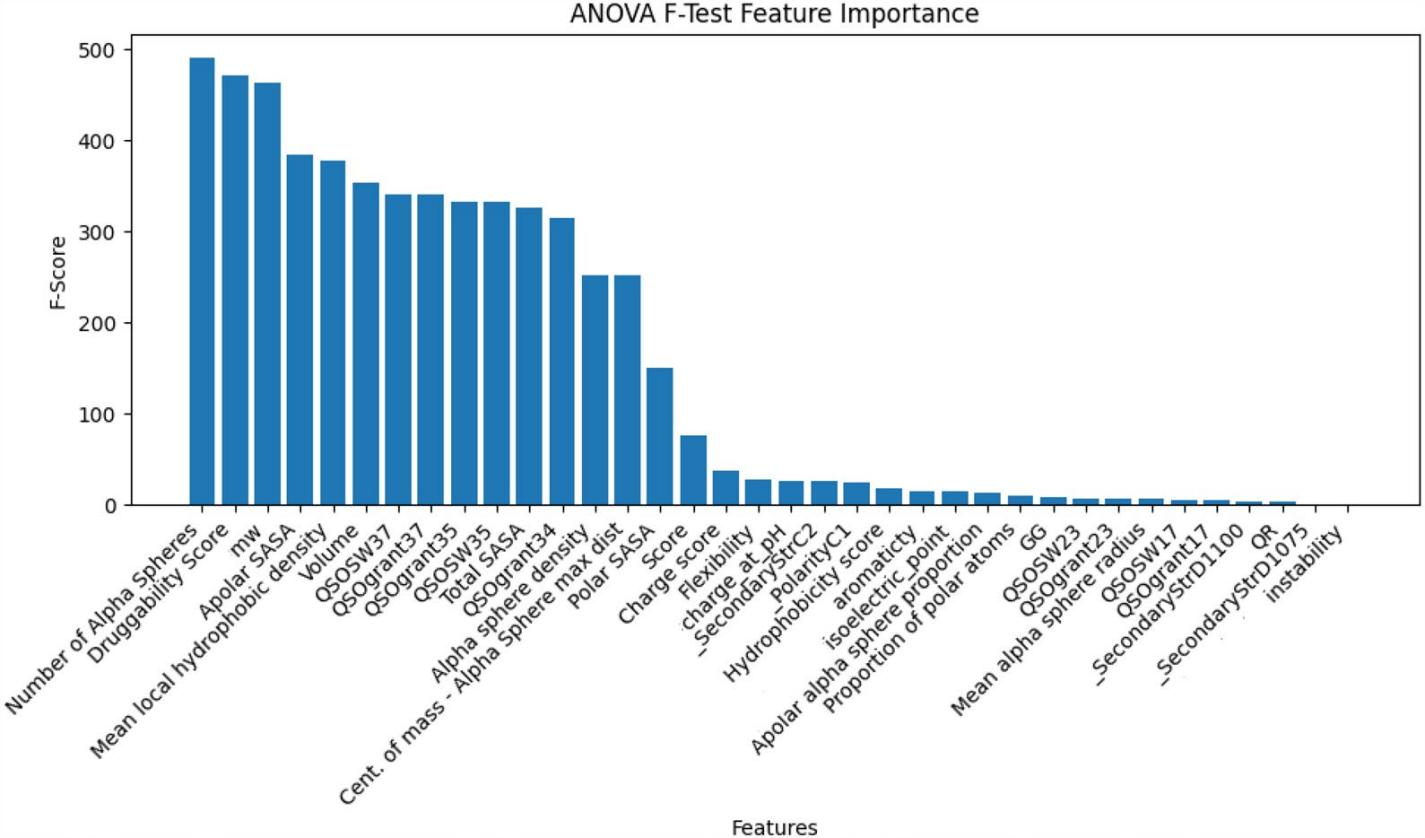
The feature significance summary is based on the merging of four selected feature sets, namely Feature Sets 1, 2, 3, and 4. Every feature displayed in the figure has been identified at least once in multimodel feature selection. The bar chart demonstrates F-Score on the y-axis and features on the x-axis. The higher the F-Score demonstrates, the higher the informative ability for machine learning models

The significance of the feature has been assessed by the utilisation of the ANOVA F-Test Feature importance methodology, which aims to gain insight into the internal workings of the model and enhance our understanding of protein allostery (Fig. 9). The pocket’s molecular weight is situated among the top three most informative characteristics. It bears a resemblance to the druggability score, albeit with a little lower significance compared to the most crucial element, namely the Number of Alpha Spheres. Additionally, it is worth noting that the inclusion of volume-based features in the analysis demonstrated a considerable level of significance (Fig. 8).

The application of the ANOVA F-test for feature importance highlighted the significance of three key features: the number of alpha spheres, the druggability score from Fpocket, and the molecular weight (MW) from Biopython. These features were ranked first, second, and third, respectively, each achieving an importance score of around 450 (Fig. 8). The frequent selection of these features across multiple methods underscores their robustness and reliability in predicting allostery in proteins (Table 9).

The ANOVA F-test for feature importance revealed the significant impact of the apolar solvent-accessible surface area (SASA), mean hydrophobic density and volume from an Fpocket with an importance score of about 375. These characteristics play essential roles in comprehending the allosteric properties of proteins. Apolar SASA is essential because it quantifies the hydrophobic surface area that is exposed to the solvent, which can affect allosteric regulation. The mean hydrophobic density indicates how hydrophobic residues are distributed throughout the pocket, which can impact the binding affinity and specificity of allosteric modulators. The pocket’s volume is a crucial characteristic that influences its ability to accept allosteric effectors of different sizes. The significant significance ratings of these traits highlight their relevance in forecasting allosteric sites and their potential influence on protein function, rendering them important predictors in the investigation of allosteric proteins.

The amino acid–based characteristics QSOgrant37, QSOSW37, QSOSW35, and QSOgrant35 from Pybiomed have shown a significance score of 350, despite their low selection frequency. These qualities are suggestive of distinct amino acid properties and sequence-based characteristics that are essential in comprehending the allostery of proteins. QSO (Quantitative structure–activity relationship) features generally encompass the spatial and electronic characteristics of amino acids in the protein structure, which might impact the protein’s dynamic behaviour and its interaction with allosteric modulators. The high importance score indicates that these descriptors based on amino acids have a crucial role in predicting allosteric locations and the overall mechanism of allosteric control, although they are infrequently picked in four feature selection approaches.

In summary, the majority of the chosen traits have low F-scores, suggesting that the task of locating an allosteric binding site has significant challenges. The potential issue that may have contributed to the diminished effectiveness of feature selection algorithms is the need for more data within the training set. While the feature set of MEF-AlloSite has limited informative features, the utilisation of a multimodel feature selection strategy has demonstrated an improvement in the performance of the algorithm for identifying binding sites.

##### The assessment of correlations among the selected features

Examining the association between certain traits is crucial for comprehending the complex mechanisms that drive protein allostery. Correlated characteristics frequently indicate the interconnections between the structural or functional components of proteins, providing insight into the intricate links between various molecular properties and their involvement in allosteric control. Significant insights into the underlying allosteric processes can be gained through the detection of correlated characteristics, enabling the observation of co-occurrence patterns or mutual effects among molecular descriptors. By comprehending these relationships, one may identify crucial structural motifs, physicochemical qualities, or sequence characteristics that collectively play a role in allosteric communication and conformational changes within the protein structure. Furthermore, investigating the relationships between characteristics helps to prioritise meaningful descriptors and remove duplicate or strongly correlated information, hence improving the predicting accuracy of computational models and making the findings easier to understand.

In addition to the inherent correlation between Fpocket and itself, there exist notable associations between 3D structural characteristics (namely, Fpocket) and attributes based on amino acids (without Fpocket). As an illustration, the Druggability score (Fpocket) exhibits six correlation values that exceed the threshold of 0.22. This type of association may facilitate comprehension of the relationship between the three-dimensional structure and amino acid–based characteristics in the context of allostery. Furthermore, the molecular weights of the cavity, as determined by Biopython, exhibit a total of fourteen positive correlation values, which span a range from 0.32 to 0.60 (Fig. 8).

The correlation matrix between selected features reveals that certain features exhibit neutral correlations with the rest of the features, with correlation scores near zero. This includes features such as GG, QR, QSOSW23, QSOSW17, QSOgrant17, QSOgrant23, _SecondaryStrD1075, and _SecondaryStrC2 from PyBioMed; flexibility, charge score, score, and mean alpha sphere radius from Fpocket; and isoelectric point and instability from Biopython. These near-zero correlations indicate that these features are relatively unique and capture distinct aspects of the protein’s properties, contributing diverse and independent information to the model.

The use of multimodel feature selection methods ensures the selection of such diverse features with low selection frequency. Without employing multiple feature selection methods, these uniquely informative features might have been overlooked despite their potential to enhance model performance. Multimodal feature selection combines the strengths of different approaches, thus capturing a broader range of relevant features that might be missed by any single method. This strategy helps identify unique features that significantly contribute to the model’s accuracy and predictive power despite their low individual selection frequencies. Consequently, the integration of multiple feature selection methods leads to the construction of a more balanced and effective feature set, improving the overall performance of the predictive models and providing deeper insights into the allosteric mechanisms of proteins.

### Case study: application of MEF-AlloSite

**Fig. 10:**
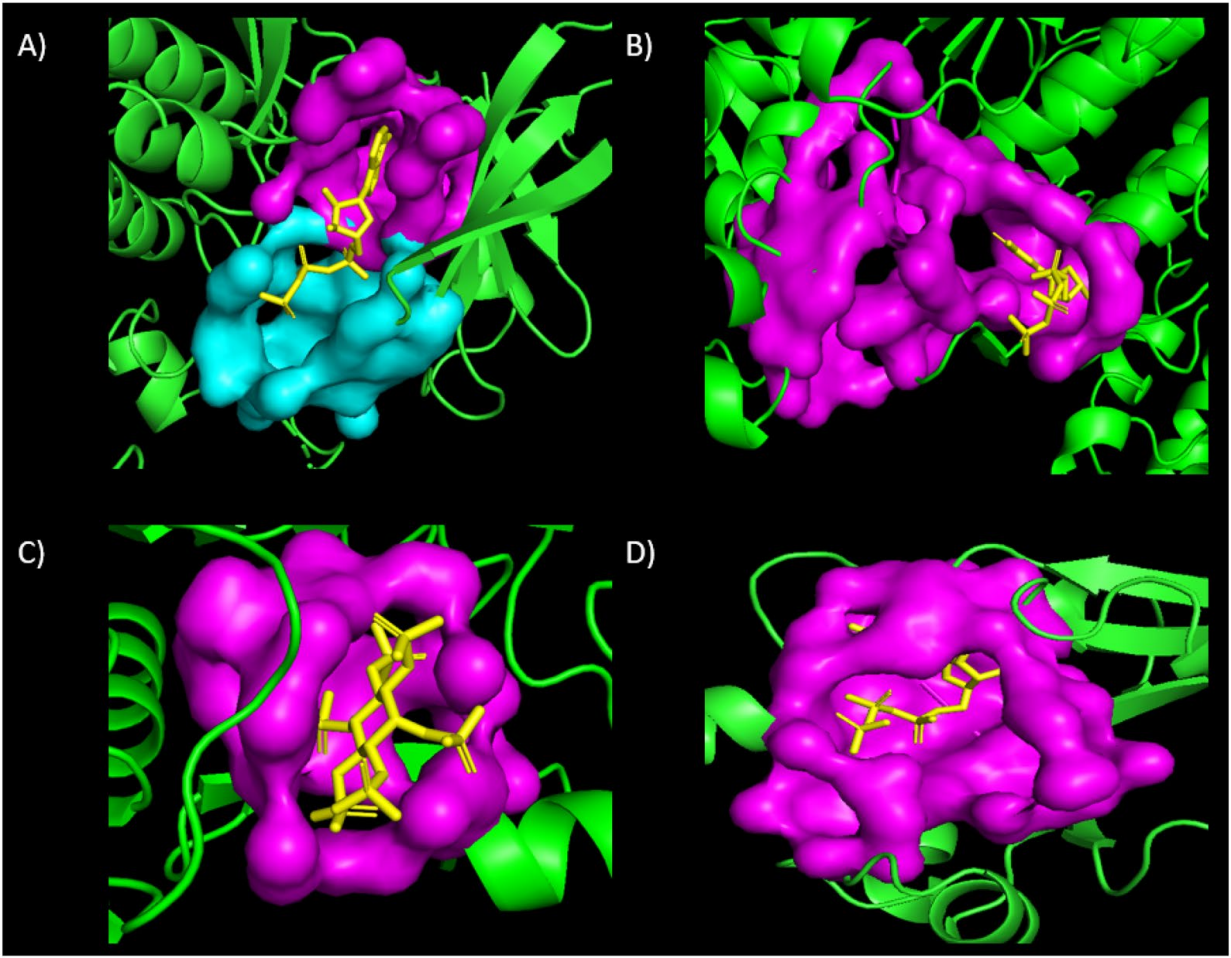
Prediction results of four examples not included in the training set. Four proteins, 2GS7, 2RIR, 3PEE, and 1COZ, are demonstrated in green on **A**, **B**, **C** and **D**, respectively. Fpocket divides an allosteric site on **A** into two different pockets, cyan and magenta. After ranking pockets by MEF-AlloSite found these pockets on the top of predictions, even if Fpocket divides and classifies them differently. The location of the allosteric ligand in yellow also demonstrates how successfully predicted pockets in magenta are predicted by MEF-AlloSite

MEF-AlloSite provides improved performance in identifying the allosteric binding site. Figure 10 demonstrates the highly ranked pockets by MEF-AlloSite in magenta and cyan and their allosteric ligands in yellow. In certain instances, the falsely predicted top one pockets are close to and even merge with the allosteric pocket (Fig. 10A). Using a different cavity detection tool may define both pockets as one, demonstrating how fairly comparing two or more allosteric binding site identification programs is challenging.

Identifying pockets is still challenging, and there is no standard to define pockets, such as pocket size. Therefore, each cavity detection tool uses unique parameters to describe pockets, which results in various cavities for the same protein. For example, Fpocket found separate pockets (Fig. 10A) even if it is one allosteric site. Also, the pockets (Fig. 10B and D) were defined as too large for allosteric ligands, while Fig. 10C demonstrates the ideal pocket, the smallest pocket size to cover the ligand. Therefore, the identification of pockets needs more standardisation.

The average number of pockets in medium-sized globular proteins having a couple of thousand atoms is 10-2048 [37]. However, the number of pockets found by cavity detection tools can significantly differ from the actual number. For example, although the actual number of pockets is around 2, the average number of predicted sites for five cavity detection tools ranges from 2.8 to 99.5 on COACH420 and HOLO4K9. The main reason for such a massive range of pocket numbers is the different pocket sizes defined by each cavity detection tool since describing larger pockets results in a few pockets on a protein [37]. The critical mistake in studies is that comparing two or more allosteric binding site models using different cavity detection tools has a strong bias to a model trained by a larger pocket size since larger pockets are highly likely to cover allosteric binding site residues and false residues. In other words, the low number of pockets because of the large size boosts the model’s allosteric binding site performance. Programs based on the same cavity detection tool should be compared to maintain consistency in labelling, pocket number, size, and location of proteins in the dataset. Therefore, MEF-AlloSite was compared to PASSer2.0 and PASSerRank to inhibit strong bias towards models trained and tested on larger pockets or lower pocket numbers [37].

## Conclusion

Over 9000 characteristics have been evaluated using feature selection techniques in order to identify the most informative feature for protein allostery. The use of Multimodel Ensemble Feature selection in MEF-AlloSite has been seen to enhance the efficacy of identifying allosteric binding sites. Furthermore, it was observed that MEF-AlloSite showed further enhancements with an increase in the number of components.

The results showed that PASSer2.0, Entire Feature Set, Ensemble Feature Selection model, and other individual models in our pipeline were considerably outperformed by MEF-AlloSite. The results of the prediction analysis revealed average accuracy values of 0.620, 0.51, and 0.452 for three test examples acquired from ADS. Furthermore, the receiver operating characteristic (ROC) area under the curve (AUC) scores were determined to be 0.866, 0.834, and 0.803 for three distinct test instances.

## Supplementary Information

Supplementary Material 1.

## Data Availability

Project Name: MEF-AlloSite. Project home page: https://github.com/yauz3/MEF-AlloSite, Dataset: ASD: https://mdl.shsmu.edu.cn/ASD/, https://github.com/yauz3/MEF-AlloSite. Operating system: Source code. Programming language: Python. Other requirements: Fpocket, Biopython, MathFeature, and PyBioMed, Fpocket: https://fpocket.sourceforge.net/, Biopython: https://biopython.org/, MathFeature: https://github.com/Bonidia/MathFeature, PyBioMed: https://github.com/gadsbyfly/PyBioMed, PASSer series: PASSer web: https://passer.smu.edu, PASSer API: https://github.com/smu-tao-group/passerAPI, PASSer2.0: https://github.com/smu-tao-group/PASSer2.0, PASSerRank: https://github.com/smu-tao-group/PASSerRank, License: GNU GPL.

## Abbreviations

MEF-AlloSite: Multimodel Ensemble Feature selection for Allosteric Site identification
ML: Machine Learning
PseAAC: Pseudo amino acid composition
CTD: Composition-Transition-Distribution
PVP: Phage virion proteins
ASD: The Allosteric Database v2.0
TPOT: The Pipeline Optimization Tool
GP: Genetic programming
AutoML: Automated Machine Learning
PS-score: Pocket Similarity Score
SVM: Support Vector Machine
MD: Molecular Dynamics
NMA: Normal-Mode-Analysis
T: Tense
R: Relaxed
PARS: Protein Allosteric and Regulatory Sites
MI: Mutual Information
SCA: Statistical Coupling Analysis
DCA: Direct Coupling Analysis
MSAs: Multiple Sequence Alignments
CTD: Composition, Transition and Distribution
GBM: Gradient boosting machine
AP: Average precision score
MW: Molecular weight
SASA: Solvent-accessible surface area
QSO: Quantitative Structure-Activity Relationship
PCA: Principal Component Analysis
tSNE: T-Distributed Stochastic Neighbour Embedding
RFE: Recursive Feature Elimination
GO: Gene Ontology
FunD: Functional Domain
MIFs: Molecular Interaction Fields
CoBDock: Consensus Blind Dock
