# Supplementary figures and images for: MEF-AlloSite: an accurate and robust Multimodel Ensemble Feature selection for the Allosteric Site identification model
Source: J Cheminform. 2024 Oct 23;16:116. doi: 10.1186/s13321-024-00882-5 (PMC11515501; doi:10.1186/s13321-024-00882-5)

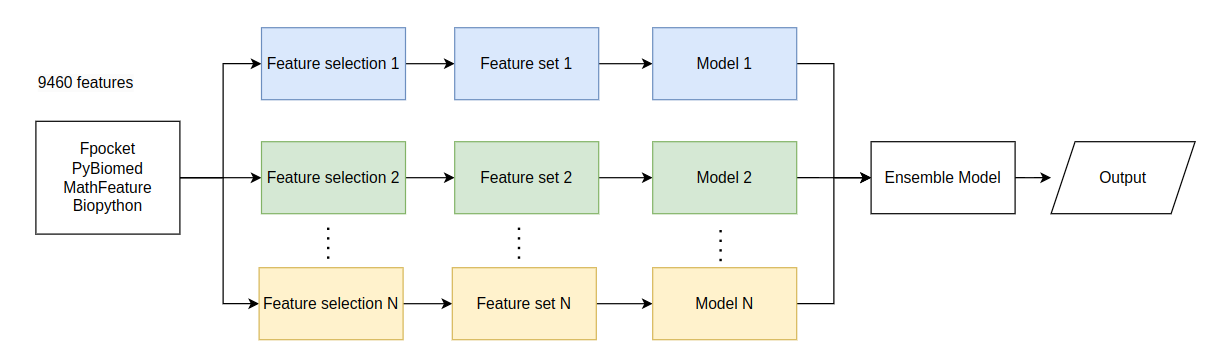

Supplement: Supplementary file 1 — Supplementary Material 1. [file 13321_2024_882_MOESM1_ESM.zip › mef_allosite_pic_1_previous.png]

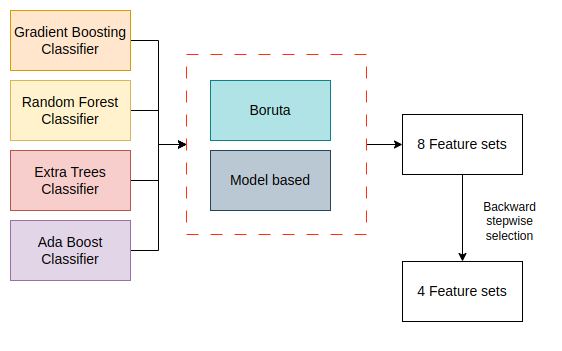

Supplement: Supplementary file 1 — Supplementary Material 1. [file 13321_2024_882_MOESM1_ESM.zip › mef_allosite_pic_2_previous.png]

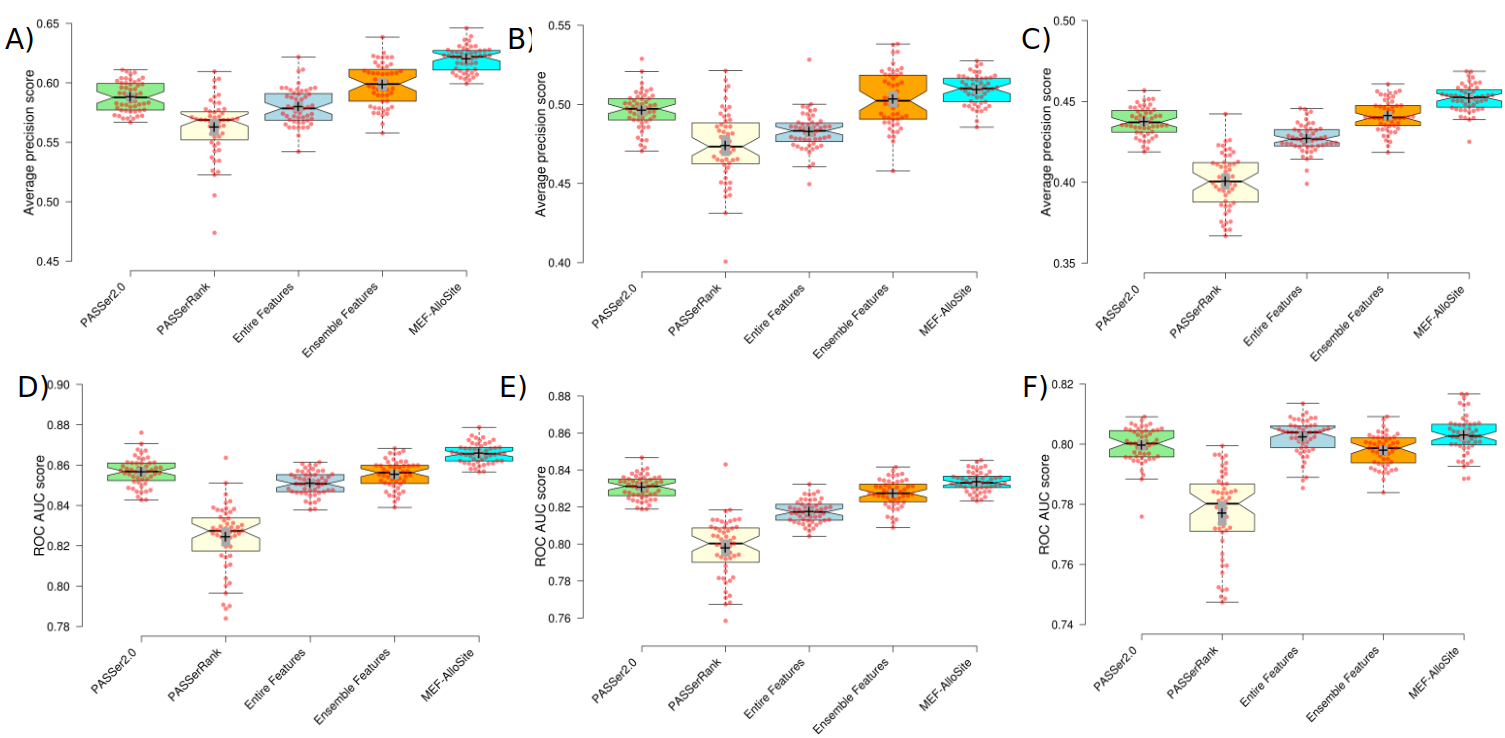

Supplement: Supplementary file 1 — Supplementary Material 1. [file 13321_2024_882_MOESM1_ESM.zip › mef_allosite_pic_3_previous.png]

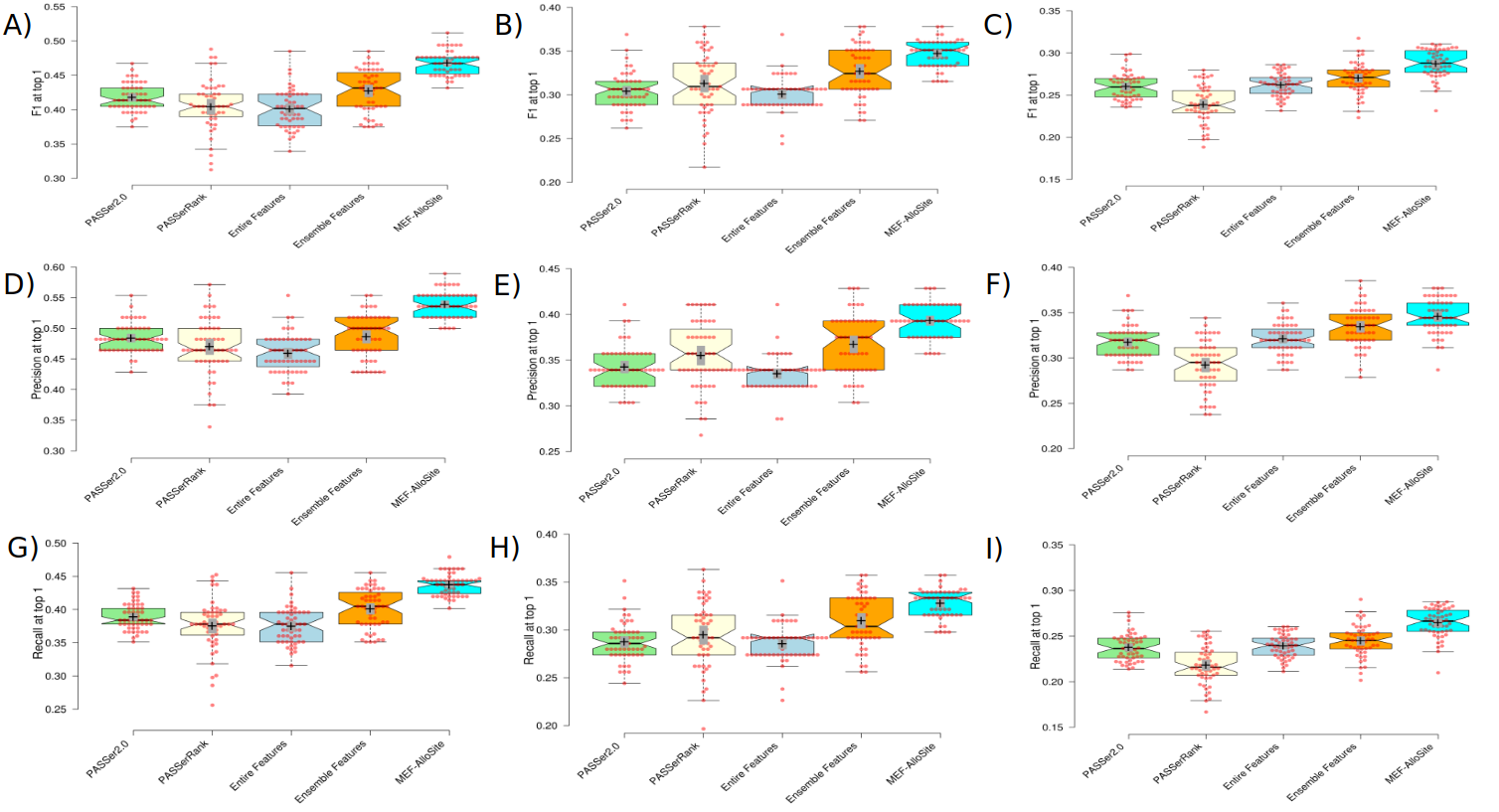

Supplement: Supplementary file 1 — Supplementary Material 1. [file 13321_2024_882_MOESM1_ESM.zip › mef_allosite_pic_4.png]

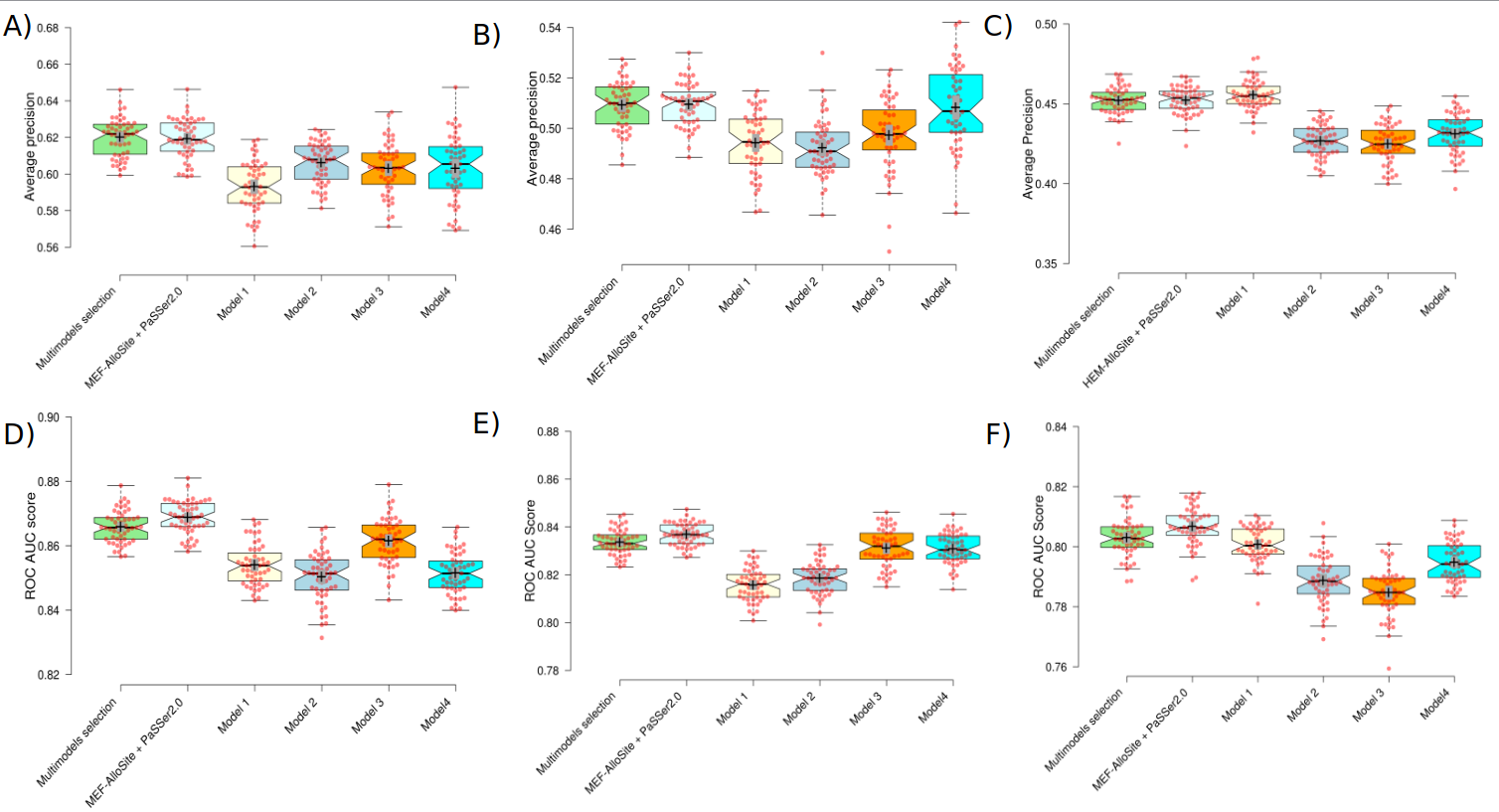

Supplement: Supplementary file 1 — Supplementary Material 1. [file 13321_2024_882_MOESM1_ESM.zip › mef_allosite_pic_5.png]

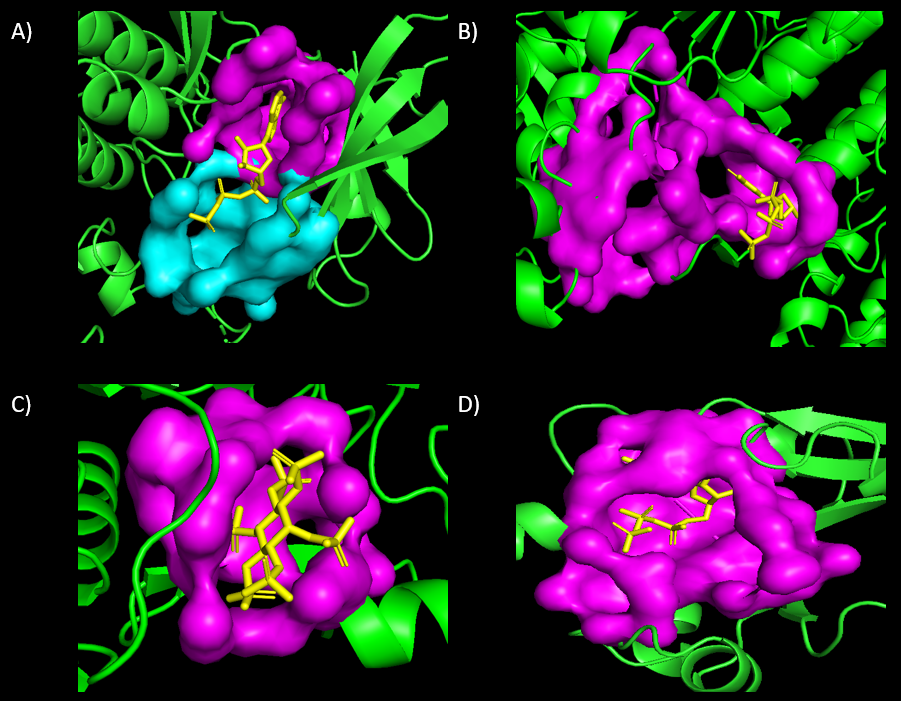

Supplement: Supplementary file 1 — Supplementary Material 1. [file 13321_2024_882_MOESM1_ESM.zip › mef_allosite_pic_8.png]

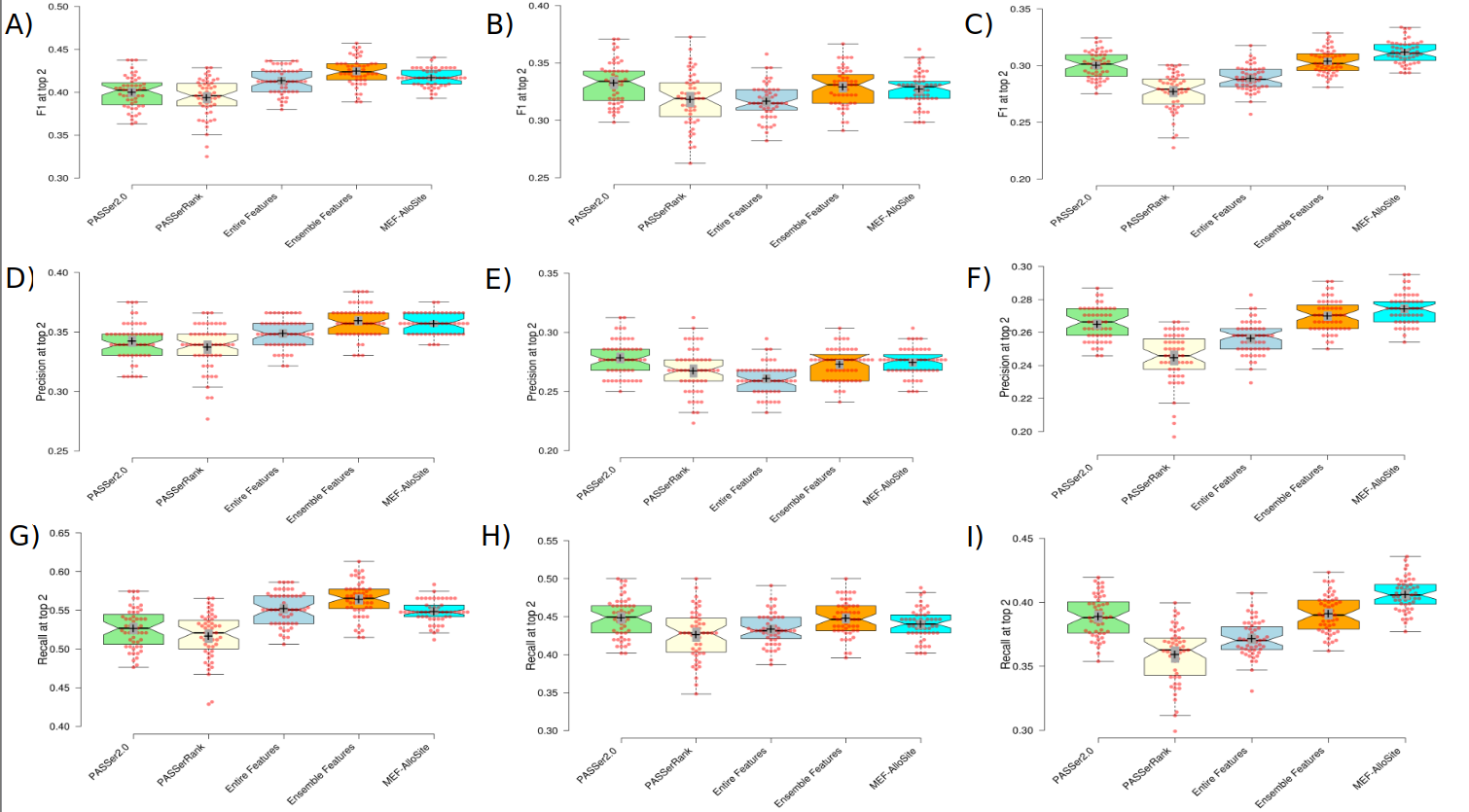

Supplement: Supplementary file 1 — Supplementary Material 1. [file 13321_2024_882_MOESM1_ESM.zip › mef_allosite_pic_9.png]

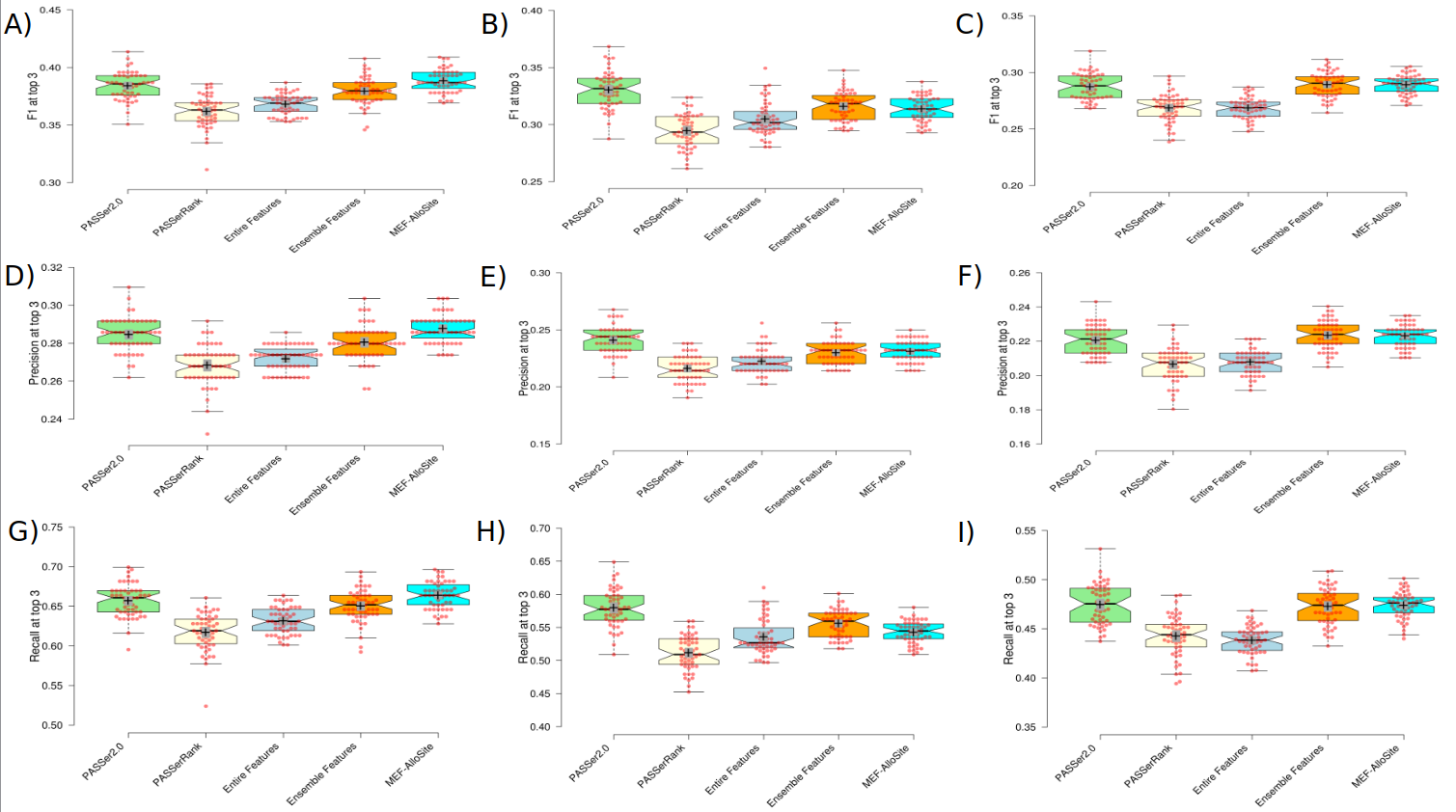

Supplement: Supplementary file 1 — Supplementary Material 1. [file 13321_2024_882_MOESM1_ESM.zip › mef_allosite_pic_10.png]

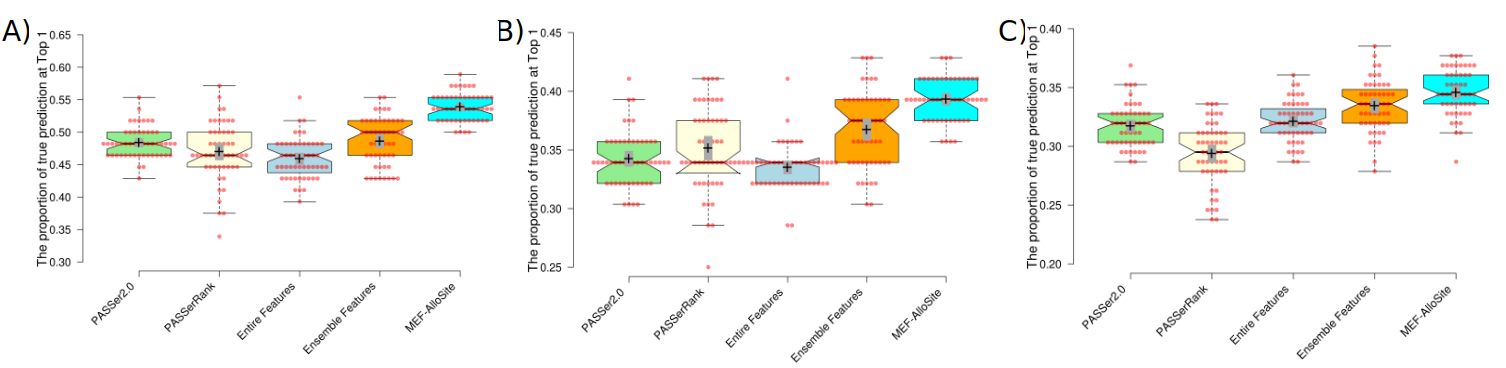

Supplement: Supplementary file 1 — Supplementary Material 1. [file 13321_2024_882_MOESM1_ESM.zip › mef_allosite_pic_11.png]

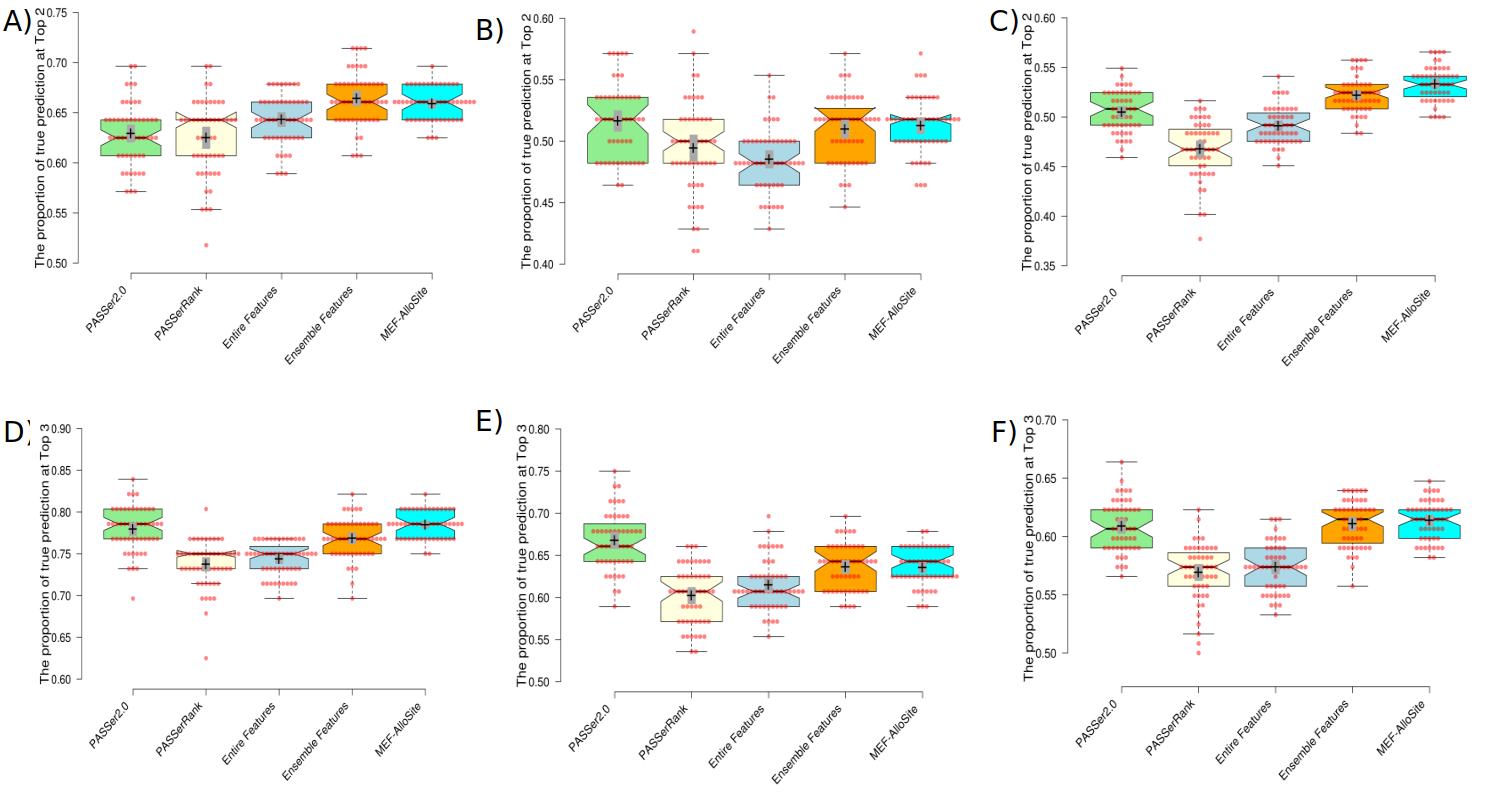

Supplement: Supplementary file 1 — Supplementary Material 1. [file 13321_2024_882_MOESM1_ESM.zip › mef_allosite_pic_12.png]

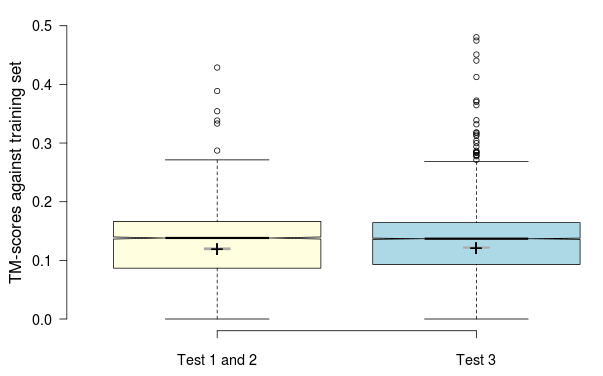

Supplement: Supplementary file 1 — Supplementary Material 1. [file 13321_2024_882_MOESM1_ESM.zip › mef_allosite_pic_13.png]

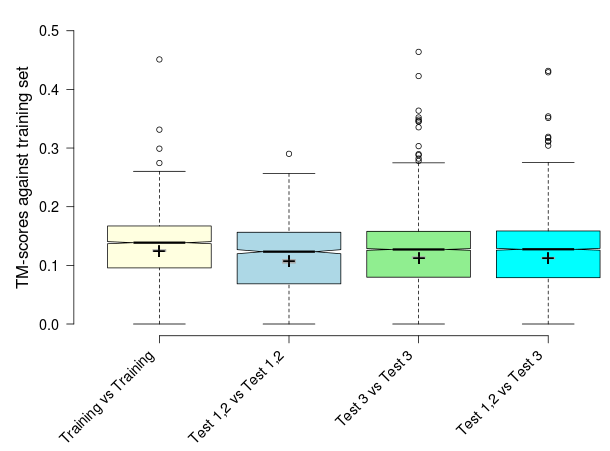

Supplement: Supplementary file 1 — Supplementary Material 1. [file 13321_2024_882_MOESM1_ESM.zip › mef_allosite_pic_14.png]

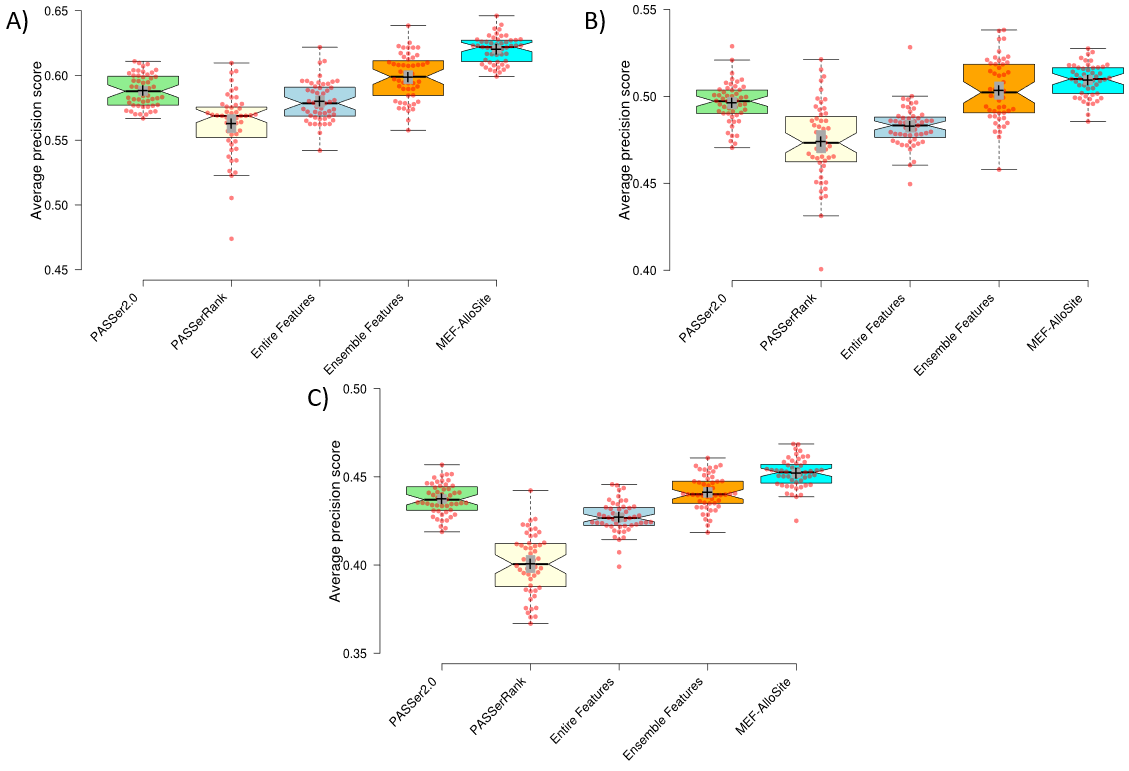

Supplement: Supplementary file 1 — Supplementary Material 1. [file 13321_2024_882_MOESM1_ESM.zip › mef_allosite_pic_3.png]

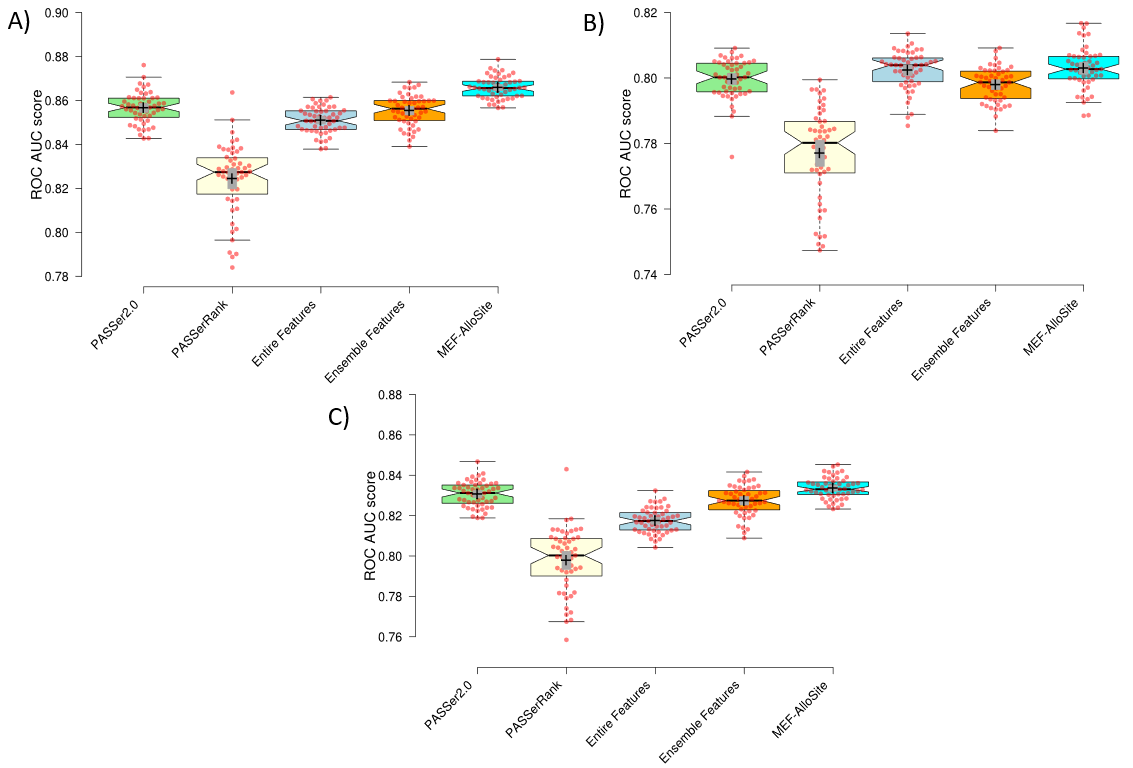

Supplement: Supplementary file 1 — Supplementary Material 1. [file 13321_2024_882_MOESM1_ESM.zip › mef_allosite_pic_3_5.png]

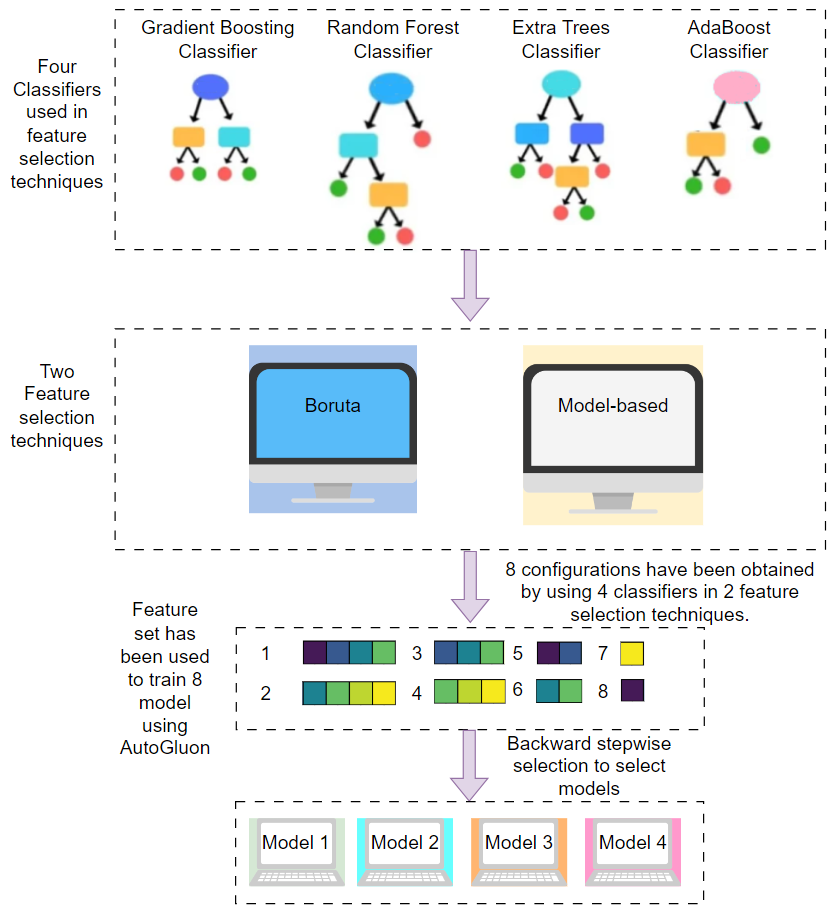

Supplement: Supplementary file 1 — Supplementary Material 1. [file 13321_2024_882_MOESM1_ESM.zip › mef_allosite_pic_2.png]

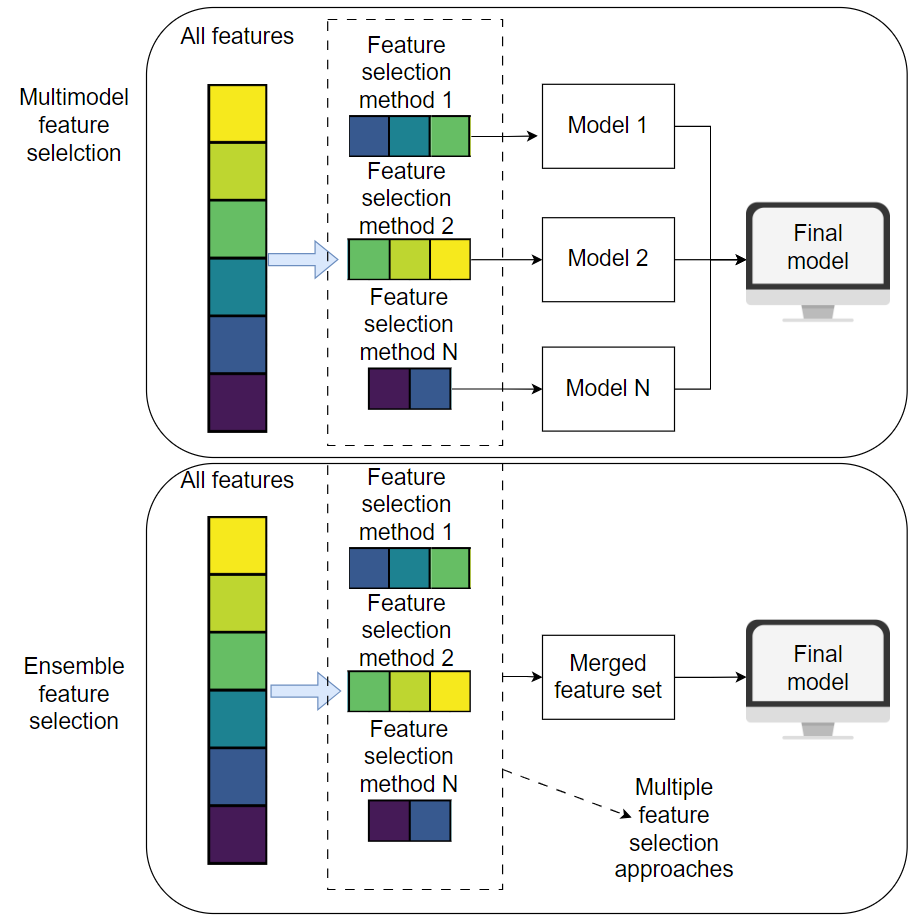

Supplement: Supplementary file 1 — Supplementary Material 1. [file 13321_2024_882_MOESM1_ESM.zip › mef_allosite_pic_0.png]

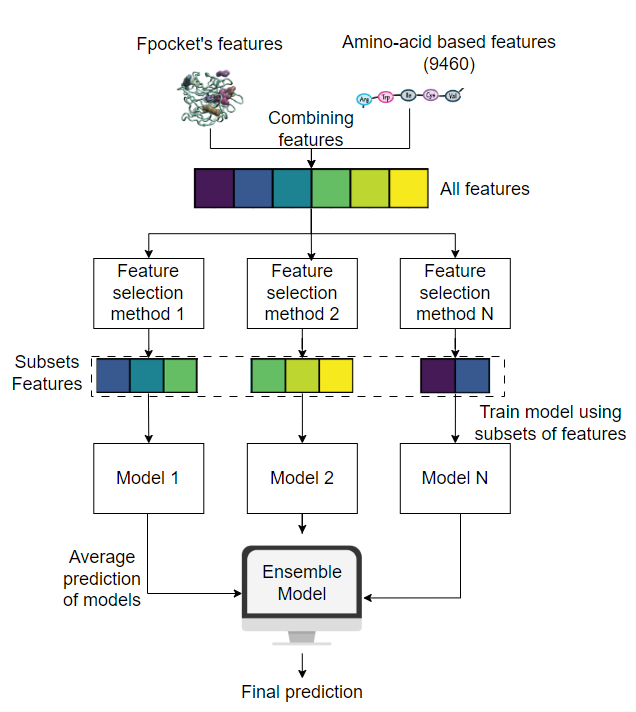

Supplement: Supplementary file 1 — Supplementary Material 1. [file 13321_2024_882_MOESM1_ESM.zip › mef_allosite_pic_1.png]

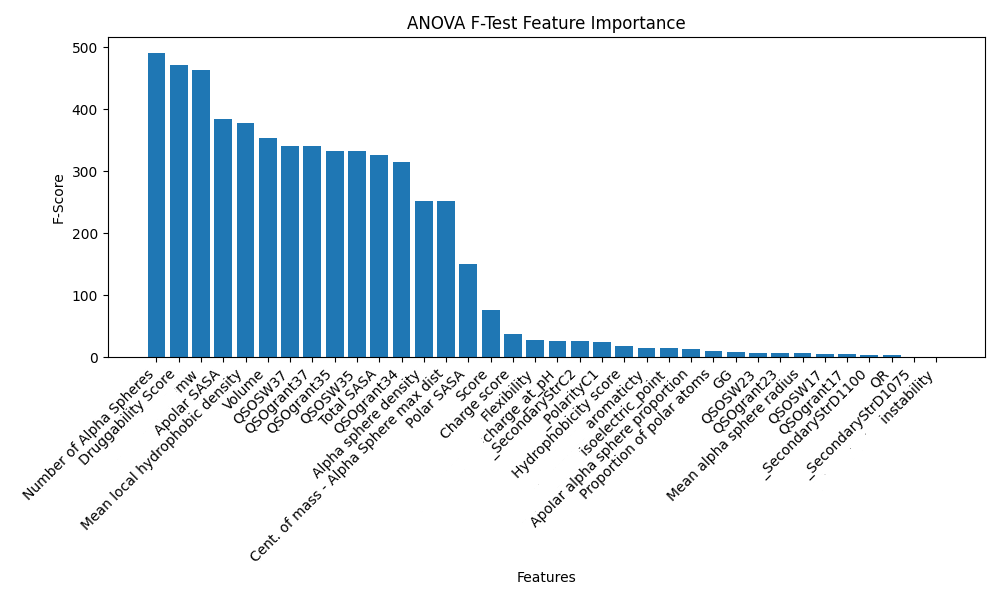

Supplement: Supplementary file 1 — Supplementary Material 1. [file 13321_2024_882_MOESM1_ESM.zip › mef_allosite_pic_7.png]

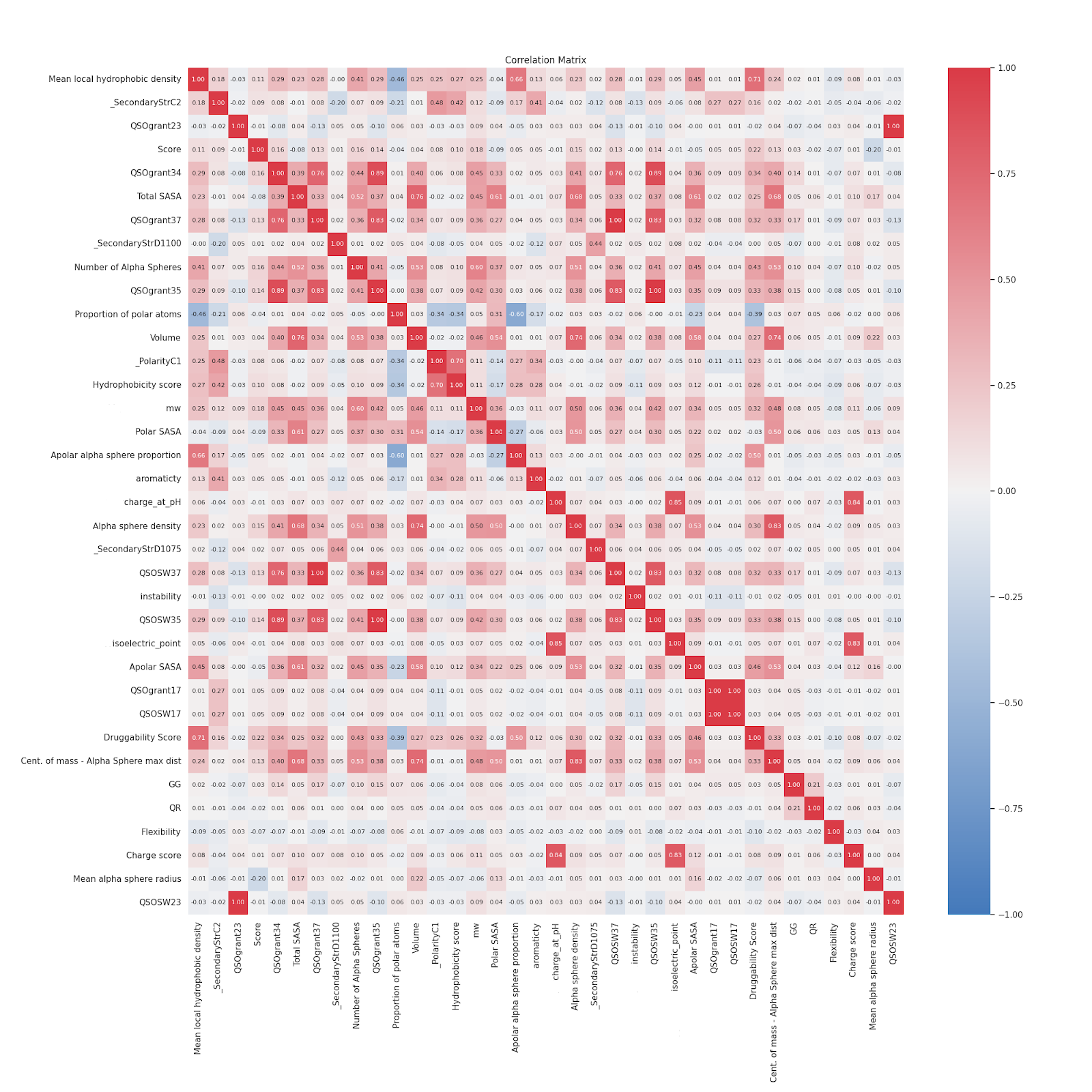

Supplement: Supplementary file 1 — Supplementary Material 1. [file 13321_2024_882_MOESM1_ESM.zip › mef_allosite_pic_6.png]
